# Environmental Monitoring of PAHs, PCBs, PCDDs, PCDFs, and PFASs in Wild Boar and Domestic Pig Tissues from Northern Italy

**DOI:** 10.3390/ani15172600

**Published:** 2025-09-04

**Authors:** Susanna Draghi, Carolina Fontanarosa, Michele Spinelli, Angela Amoresano, Stefano Materazzi, Roberta Risoluti, Dalia Curci, Giulio Curone, Petra Cagnardi, Francesco Arioli, Federica Di Cesare

**Affiliations:** 1 Department of Veterinary Medicine and Animal Sciences, University of Milan, Via dell’Università 6, 26900 Lodi, Italy; susanna.draghi@unimi.it (S.D.); dalia.curci@unimi.it (D.C.); giulio.curone@unimi.it (G.C.); petra.cagnardi@unimi.it (P.C.); federica.dicesare@unimi.it (F.D.C.); 2 Istituto Nazionale Biostrutture e Biosistemi, I.N.B.B., 00136 Rome, Italy; carolina.fontanarosa@unina.it (C.F.); michele.spinelli@unicampania.it (M.S.); angela.amoresano@unina.it (A.A.); stefano.materazzi@unina.it (S.M.); roberta.risoluti@uniroma1.it (R.R.); 3 Dipartimento di Scienze Chimiche, Università degli Studi di Napoli Federico II, Strada Comunale Cinthia, 26, 80126 Napoli, Italy; 4 First Division of Nephrology, Department of Translational Medical Sciences, School of Medicine, University of Campania “Luigi Vanvitelli”, Via M. Longo 50, 80138 Naples, Italy; 5 Department of Chemistry, Sapienza University of Rome, Piazzale Aldo Moro 5, 00185 Rome, Italy

**Keywords:** biomonitoring, ecotoxicology, environmental contaminants, terrestrial wild mammals, contaminants

## Abstract

This study investigated the bioaccumulation patterns of polycyclic aromatic hydrocarbons (PAHs), polychlorinated biphenyls (PCBs), and per- and polyfluoroalkyl substances (PFASs) in the liver and muscle tissues of wild boars (*n* = 39) and domestic pigs (*n* = 38) from Northern Italy. This research addressed a critical gap in our understanding of how different ecologies and diets influence the uptake of persistent organic contaminants in two closely related species, one domestic and one wild. Significant differences in contaminant profiles were observed, largely attributable to distinct exposure routes and feeding behaviors. Wild boars displayed different quantities and families of environmental contaminants, with higher PCB levels in muscle and PFASs in liver. Conversely, domestic pigs exhibited markedly higher PAH concentrations, primarily linked to contaminated feed in controlled agricultural settings. The liver consistently demonstrated a central role in toxicant retention across both species. Notably, concentrations of several regulated PFAS compounds in both wild and farmed animals exceeded EU maximum levels (sum of PFOS, PFOA, PFNA, and PFHxS: 1.3 µg/kg), raising significant food safety concerns. These findings underscore the critical need for continuous environmental biomonitoring, stricter control of contaminant sources in agriculture, and updated risk assessments for both wild and domestic meat products to protect animal welfare and human health.

## Simple Summary

Our research compared levels of harmful chemicals, including polycyclic aromatic hydrocarbons (PAHs), polychlorinated biphenyls (PCBs), and per- and polyfluoroalkyl substances (PFASs), in wild boars and domestic pigs from Northern Italy. We found that wild boars accumulated compounds from each family of contaminants investigated, especially in their livers and muscles. Domestic pigs, however, had higher levels of PAHs, particularly in their livers, likely due to contaminated feed. Both species showed high levels of chemicals in their livers, indicating this organ’s importance in processing toxins. Worryingly, many PFAS levels in both wild and farmed meat exceeded safe limits set by the EU, posing a risk to people consuming these products. This highlights the urgent need for ongoing environmental checks, better control over contaminant sources in farming, and updated safety guidelines to protect both animals and human health.

## 1. Introduction

Persistent organic pollutants (POPs) or, more generally, persistent bioaccumulative toxic substances (PBTs), including dioxins, furans classified as polychlorinated dibenzo-p-dioxins (PCDDs) and polychlorinated dibenzofurans (PCDFs), polychlorinated biphenyls (PCBs), polycyclic aromatic hydrocarbons (PAHs), and per- and polyfluoroalkyl substances (PFASs), pose a serious global environmental and health concern due to their resistance to degradation, ability to bioaccumulate, potential for long-range transport, and toxicity [1,2]. These substances originate from both natural and anthropogenic sources, such as industrial processes, combustion, and waste incineration [3,4]. Dioxins and furans are among the most toxic contaminants and can cause endocrine disruption, reproductive impairments, and developmental toxicity even at trace concentrations [5]. They are typically released during combustion events, including industrial emissions, burning of waste, and forest fires [6]. PAHs are another widespread group of contaminants produced mainly by the incomplete combustion of organic materials like coal, petroleum, wood, and tobacco [7]. Due to their presence in air, water, and soil, PAHs can easily enter the food chain, accumulating in fatty tissues. Their genotoxic, carcinogenic, and immunosuppressive effects make their occurrence in food-producing animals particularly relevant to public health [8]. Similarly, although PCBs have been banned for decades in many countries, they continue to persist in the environment and remain detectable in soil, sediments, wildlife, and human tissues [9]. Once widely used in electrical equipment and industrial applications, PCBs are linked to cancer, immune system suppression, reproductive toxicity, and neurodevelopmental disorders, especially in children [10,11]. As a large group of synthetic organofluorine compounds, PFASs have come under increasing scrutiny due to their widespread use in consumer and industrial products. Known as “forever chemicals” for their exceptional persistence [12], PFASs are associated with liver damage, thyroid disruption, developmental effects, immune dysfunction, and elevated cancer risk [13,14]. Animals’ exposure to these contaminants occurs through ingestion of polluted feed, soil, or water; inhalation of contaminated air; and dermal contact [15,16,17]. In wildlife, exposure is often related to proximity to landfills, industrial zones, and agricultural areas [18]. Domestic animals, particularly those raised in outdoor or semi-extensive systems, are equally vulnerable when grazing in contaminated environments [19]. The accumulation of environmental contaminants in animal tissues not only endangers animals’ health and productivity but also raises concerns about food safety and the transfer of contaminants to humans through consumption of meat and other animal products [20,21].

Biomonitoring plays a crucial role in assessing contaminant levels and identifying sentinel species in environmental toxicology [22,23]. Wild boars, due to their omnivorous diet, broad foraging range, and frequent interaction with human-altered environments, are considered valuable bioindicators of terrestrial contamination [24]. Their tissue profiles provide key insights into the presence and distribution of environmental contaminants, supporting ecological risk assessments and public health strategies [25]. The One Health approach emphasizes the interconnection between environmental integrity, animal health, and human well-being, advocating for integrated surveillance and management. Alongside this, the concept of One Toxicology promotes harmonized toxicological assessments across species to improve risk predictions and cross-species comparisons [26]. However, the systematic comparison of pollutant burdens in wild boar populations across different regions and countries remains limited. For instance, while studies have been conducted in regions like Central Europe and the Iberian Peninsula, a coordinated effort to compare specific legacy and emerging contaminants, such as PFASs and PCBs, is often lacking. Such comparative studies are essential for understanding the global distribution of these contaminants and for establishing baseline data for different ecosystems [27,28]. Monitoring legacy and emerging POPs in both wild and domestic species serves as an early warning system, enabling timely interventions to protect ecosystems, food safety, and public health. Integrating these frameworks allows for a more comprehensive understanding of pollutant dynamics and supports evidence-based policy development [29].

In this context, the present study aimed to assess and compare the levels of PAHs, PCBs, PFASs, PCDDs and PCDFs in liver and muscle tissues of wild boars and domestic pigs from the same geographical area of Northern Italy. Comparing these two species within a shared ecological setting allows for a deeper understanding of contaminant transfer in terrestrial food webs and provides crucial data for refining risk assessment models. In fact, in rural and peri-urban areas, the proximity between wildlife and livestock increases the potential for cross-species contaminant transfer via shared resources and habitats [13]. This overlap underscores the need and the importance of including both domestic and wild species in environmental surveillance programs.

## 2. Materials and Methods

### 2.1. Sample Collection

For this study, we collected liver and muscle samples (100 g each) from 77 animals, with ethical approval obtained from the Animal Welfare Organization of Milan University (authorization no. 26_2022/02-04-2022). Specifically, liver and muscle tissues were collected from 39 wild boars (*Sus scrofa*) culled during official control programs and from 38 domestic pigs (*Sus scrofa domesticus*) raised outdoors in the same geographical area of Northern Italy. All samples were collected in glass tubes to avoid additional contaminations from plasticizers, immediately refrigerated, transported to the laboratory, and then frozen at −20 °C for later analysis. The animals were selected to represent comparable environmental exposure conditions, and wild boar killing points were georeferenced. Wild boar samples were obtained during the hunting season (October–December) at hunting meat processing plants, with animal selection guided by established hunting and monitoring plans rather than specific sampling criteria. We also recorded morphometric measures (weight, total trunk length, chest circumference, and height at the withers) and estimated age via dental eruption. To avoid differences caused by seasonality and to standardize the sampling, the domestic pigs were also sampled during the same period.

Considering that the study area, being a rural area, should be free of PFAS contamination and that there are no similar studies in the literature, we assessed its presence in half of the subjects (20 domestic pigs and 20 wild boars).

### 2.2. Analysis of PAHs, PCBs, and PCDDs and PCDFs

#### 2.2.1. Chemicals and Standards

All solvents were purchased from Sigma-Aldrich (St. Louis, MO, USA). Certified standard mixes of PAHs, PCBs, and dioxins (10 µg/mL) were acquired from Agilent (Santa Clara, CA, USA) and diluted in toluene (for PAHs and dioxins) or isooctane (for PCBs). Calibration curves were prepared by serial dilution in the range 0.5–100 µg/L and stored at −20 °C.

The complete list of the compounds included in the method can be found for PAHs, PCBs, PCDDs and PCDFs in the Supplementary Materials (Tables S1 and S2).

#### 2.2.2. Sample Preparation

Tissues were weighed, ground in liquid nitrogen, and extracted using a 1:1 (*v*/*v*) hexane–acetone mixture. Samples were mechanically shaken for 2 min and sonicated for 30 min. A second extraction step was performed by adding fresh solvent and repeating the agitation and sonication. The organic phase was separated by centrifugation (10 min, 10,000 rpm), evaporated under nitrogen flow, and reconstituted in 100 µL of hexane.

#### 2.2.3. GC-MS/MS Analysis

Quantitative analysis of PAHs, PCBs, and dioxins was performed using an Agilent 7000C GC-MS/MS system equipped with a 7693 Autosampler and DB-5MS UI capillary column (60 m × 0.25 mm × 0.25 µm). Helium was used as carrier gas and nitrogen as collision gas. Dedicated chromatographic programs were applied for each class of compounds due to differing thermal properties. Data was acquired in multiple reaction monitoring (MRM) mode, and peak integration was carried out using Agilent Mass Hunter Quantitative Analysis software (version B.07.00). External standard quantification was used.

#### 2.2.4. Method Validation

Linearity, matrix effects, and limits of detection (LODs) and quantification (LOQs) were evaluated through matrix-matched calibration curves. The method was linear (R^2^ ≥ 0.999) for all analytes. LODs were calculated based on repeated blank spiking (*n* = 10) and using the formula LOD = 3.3 × (standard deviation/slope). Recovery rates were also assessed.

Specific chromatographic conditions for PAHs, PCBs, PCDDs, and PCDFs are reported in the Supplementary Materials.

### 2.3. Analysis of PFAS

#### 2.3.1. Chemicals and Standards

All standards were purchased from Wellington (Boston, MA, USA). All the solutions and solvents were of the highest available purity and were suitable for LC–MS/MS analysis and purchased from J. T. Baker (Phillipsburg, NJ, USA). The mixture of labeled standards used for the calibration curves had a concentration of 200 µg/L and included the following standards: Perfluoro-n-[1,2,3,4-13C4]butanoic acid 2 µg/mL, Perfluoro-n-[1,2-13C2]hexanoic acid 2 µg/mL, Perfluoro-n-[1,2,3,4-13C4]octanoic acid 2 µg/mL, Perfluoro-n-[1,2,3,4,5-13C5]nonanoic acid 2 µg/mL, Perfluoro-n-[1,2-13C2]decanoic acid 2 µg/mL, Perfluoro-n-[1,2-13C2]undecanoic acid 2 µg/mL, Perfluoro-n-[1,2-13C2]dodecanoic acid 2 µg/mL, Sodium perfluoro-1-hexane [18O2]sulfonate 2 µg/mL, Sodium perfluoro-1-[1,2,3,4-13C4]octanesulfonate 2 µg/L. The stock solutions were stored at −20 °C until the analysis. The complete list of the PFASs included in the method can be found in Table S4.

#### 2.3.2. Sample Preparation and LC-MS/MS Analysis

The samples were analyzed under the same conditions as the standards. The obtained instrument response was compared to the calibration curve to determine the analyte concentration in the sample and recovery.

Samples were fortified and extracted as reported before [30], and (parXXX) 3 μL was analyzed using a AB-sciex 5500 QTRAP^®^ (Framingham, MA, USA) system with a HPLC chromatography system Exion LC™ (Framingham, MA, USA). The column used was Kinetex 5 µm C18 100 Å (Torrance, CA, USA). The mobile phase was generated by mixing eluent A (0.1% Formic Acid in water) and eluent B (0.1% Formic Acid methanol), and the flow rate was 0.200 mL/min. The chromatographic gradient was 20% B for 2 min, to 90% in 2 min, held for 6 min, then returned to 20% in 1 min. Tandem mass spectrometry was performed using a Turbo VTM ion source (Framingham, MA, USA) operated in negative ion mode, and the multiple reaction monitoring (MRM) mode was used for the selected analytes. Tables S4 and S5 provide a list of precursor ions, product ions, collision energy, and polarity. Skyline software (version 21.2) was used for peak integration.

#### 2.3.3. Quantification and Validation

Calibration curves were generated by plotting peak area versus analyte concentration (µg/L), and linear regression was applied. The coefficient of determination (R^2^) was greater than 0.99 for all PFAS compounds analyzed (Tables S4 and S5).

#### 2.3.4. Spike and Recovery Calculation

For the evaluation of recovery and matrix effects, all samples were fortified prior to extraction with two isotopically labeled standards: Perfluoro-n-(1,2,3,4,5-13C5) nonanoic acid (1.2 mL; 50.0 µg/mL) and Sodium perfluoro-1-(1,2,3,4-13C4) octane sulfonate. Each sample was spiked with 25 µL of a 50.0 µg/mL standard solution, assuming a final volume of 200 µL. A 3 μL aliquot was injected for LC-MRM-MS analysis, and all measurements were performed in triplicate. Recovery, calculated by integrating the peak areas of the internal standards in individual samples, ranged from 90% to 97%.

### 2.4. Statistical Analysis

Statistical analysis was conducted using GraphPad InStat 8 software. Initially, data were categorized by species (domestic pigs = 38 and wild boar = 39). Pairwise comparisons were then made, first between liver and muscle tissues within each species, and subsequently between the liver of domestic pigs and wild boars, and similarly, between the muscle of both species. The Shapiro–Wilk normality test was performed, and as the data were found to be non-normally distributed, a non-parametric statistical test (Mann–Whitney test) was applied for the comparisons between the two categories.

## 3. Results

During the study, samples were also analyzed for the presence of PCDDs and PCDFs, which were never detected in the specimens. The compounds reported in the figures and tables include only those that were both detected and quantified above the limit of quantification (LOQ). Compounds that were below the LOQ or not detected are provided in the Supplementary Materials.

### 3.1. PAHs Detected in Wild Boar and Domestic Pig Tissues

**Figure 1:**
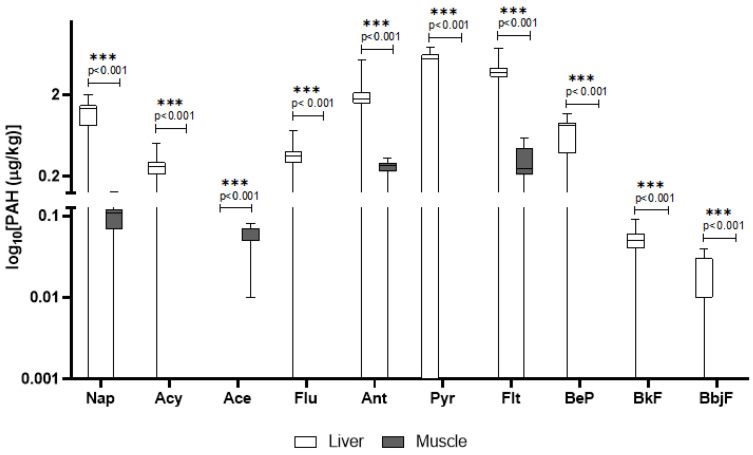
Detected concentrations of PAHs in wild boar liver and muscle. The *y*-axis is plotted on a log_10_ scale. Values are expressed in μg/kg. Only compounds above the LOQ are shown. Nap = naphthalene; Acy = Acenaphthylene; Ace = Acenaphthene; Flu = fluorene; Ant = Anthracene; Pyr = pyrene; Flt = fluoroanthene; BeP = benzo[e]pyrene; BfK = benzo[k]fluoroanthene; BbjF = benzo[b,j]fluoroanthene.

In wild boars, several PAHs were detected, with higher concentrations generally found in the liver compared to the muscle. Notably, pyrene, fluoranthene, and anthracene showed the highest mean concentrations in the liver. Statistically significant differences (*p* < 0.001) were found between tissues for most compounds (Figure 1). However, detection frequency (DF) varied: for instance, pyrene had a DF of 64.9%, whereas anthracene was detected in over 89% of liver samples. Compounds such as benzo_b,j_fluoranthene and benzo_k_fluoranthene had lower concentrations and DFs (78%) (Table 1). The other compounds of the PAH class that were screened for but not detected are reported in the Tables S4 and S5.

**Figure 2:**
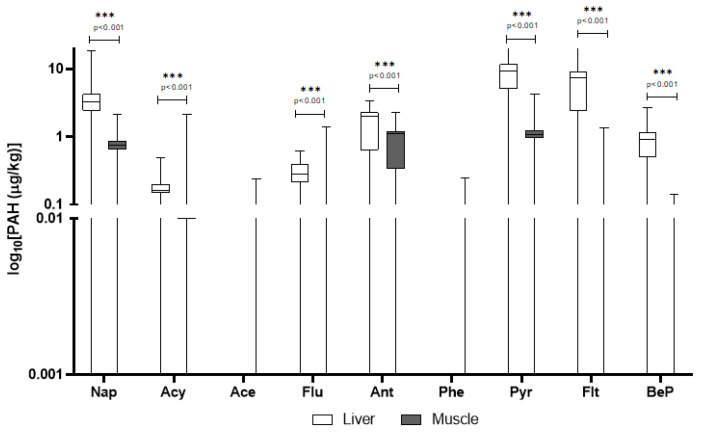
Detected concentrations of PAHs in domestic pigs’ liver and muscle. The *y*-axis is plotted on a log_10_ scale. Values are expressed in μg/kg. Only compounds above the LOQ are shown. Nap = naphthalene; Acy = Acenaphthylene; Ace = Acenaphthene; Flu = fluorene; Ant = Anthracene; Phe = phenanthrene; Pyr = pyrene; Flt = fluoroanthene; BeP = benzo[e]pyrene.

In domestic pigs, pyrene, anthracene, and fluoranthene were again prominent, with pyrene showing particularly high levels in the liver (mean: 9.29 μg/kg) and a DF of 81%. Interestingly, naphthalene had much higher concentrations in domestic pigs’ tissues than in wild boar, with a DF > 83%. Several PAHs detected in domestic pigs were absent or below LOQ in wild boar (e.g., phenanthrene in muscle) (Table 2). In Figure 2 are reported only PAHs quantified above LOQ in liver and muscle of domestic pigs.

**Figure 3:**
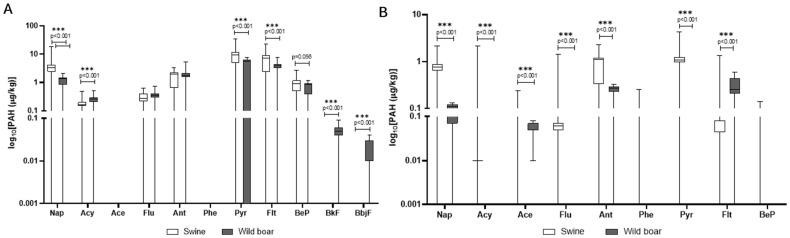
Comparison of PAH concentrations between wild boar and domestic pigs in liver (**A**) and muscle (**B**). The *y*-axis is plotted on a log_10_ scale. Values are in μg/kg. Only compounds above the LOQ are shown. Nap = naphthalene; Acy = Acenaphthylene; Ace = Acenaphthene; Flu = fluorene; Ant = Anthracene; Pyr = pyrene; Flt = fluoroanthene; BeP = benzo[e]pyrene; BfK = benzo[k]fluoroanthene; BbjF = benzo[b,j]fluoroanthene.

When comparing species (Figure 3A,B), domestic pigs’ tissues showed markedly higher concentrations of most PAHs, particularly in the liver, where pyrene and fluoranthene levels exceeded those observed in wild boar. In contrast, wild boar liver exhibited a greater variety of PAHs, despite generally lower concentrations. Anthracene was detected in both species at comparable frequencies, yet concentrations in domestic pigs’ muscle were significantly higher, indicating possible differences in metabolic processing or source exposure. Interestingly, some PAHs such as phenanthrene were detected only in domestic pigs’ muscle. Overall, tissue-specific accumulation patterns differed across species, with wild boar tending to accumulate PAHs preferentially in the liver, while domestic pigs displayed a more even distribution between muscle and liver.

### 3.2. PCBs Detected in Wild Boar and Domestic Pig Tissues

**Figure 4:**
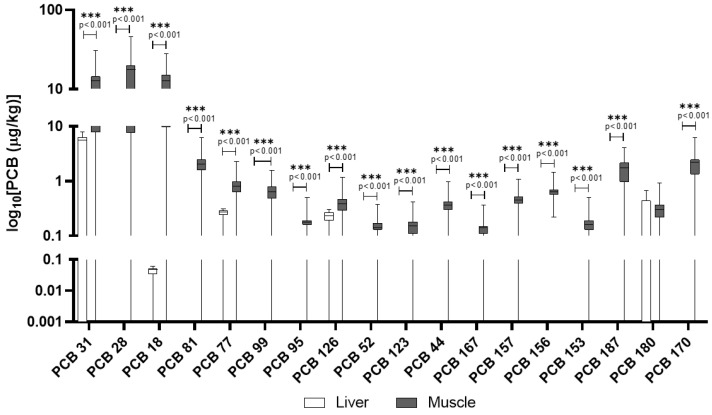
PCBs detected in wild boar muscle and liver. The *y*-axis is plotted on a log_10_ scale. Values are reported in µg/kg. Only compounds above the LOQ are shown.

In wild boars, muscle tissue showed a broader profile of PCB congeners compared to the liver, where only a few were quantifiable (Table 3). For example, PCB 31, PCB 28, and PCB 18 reached substantial concentrations in muscle (mean up to 15.9 μg/kg), with detection frequencies consistently above 75% and in some cases, over 95% (e.g., PCB 44, 123, 156). Most dioxin-like PCBs (e.g., PCB 126) were also detected in muscle but rarely in liver. In Figure 4 are reported PCB congeners quantified in liver and muscle of wild boars, while the additional PCB congeners included in the screening but not detected are reported in the Table S6.

**Figure 5:**
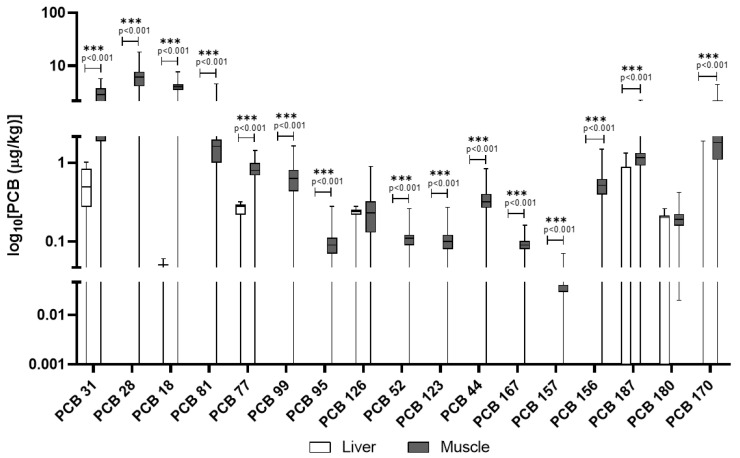
PCBs detected in domestic pigs’ muscle and liver. The *y*-axis is plotted on a log_10_ scale. Values are reported in µg/kg. Only compounds above the LOQ are shown.

Domestic pigs had a contrasting pattern (Figure 5). PCB accumulation was more evenly distributed between liver and muscle. Notably, PCB 180 and PCB 153 were detected in 100% and 89% of muscle samples, respectively, with lower but measurable levels in the liver (Table 4). Table S7 lists additional PCB congeners that were screened in domestic pigs but not detected in any of the samples.

**Figure 6:**
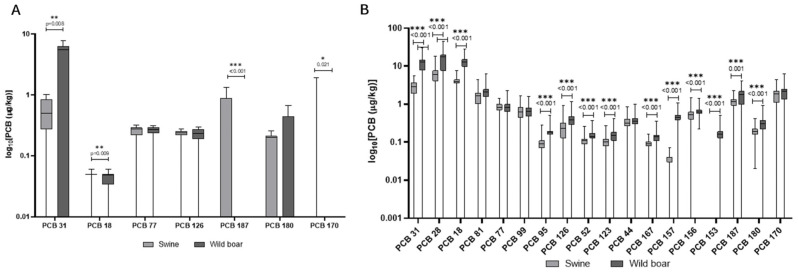
Comparison of concentrations of PCBs in the liver (**A**) and muscle (**B**) of wild boars and domestic pigs. The *y*-axis is plotted on a log_10_ scale. The data are reported in μg/kg. Only compounds above the LOQ are shown.

In Figure 6A,B, the comparison of concentrations of PCBs in the liver and muscle of wild boars and domestic pigs is reported. Unlike PAHs, PCB levels were generally higher in wild boar muscle compared to domestic pigs, with several congeners (e.g., PCB 28, PCB 31) showing mean concentrations more than double those in domestic animals. The diversity of PCB congeners was greater in wild boars, particularly in muscle tissue. Domestic pigs, however, showed a more balanced distribution of PCBs across tissues, and some congeners such as PCB 180 and PCB 153 were consistently detected in both liver and muscle. Dioxin-like PCBs (e.g., PCB 126, PCB 77) were found at higher frequencies and concentrations in wild boar muscle than in liver, whereas in domestic pigs, levels were generally lower and more evenly distributed.

### 3.3. PFASs Detected in Wild Boar and Domestic Pig Tissues

All PFASs analyzed were detected in 100% of samples, both in wild boar and domestic pigs, for all tissue types, indicating a ubiquitous contamination profile (Table 5).

**Figure 7:**
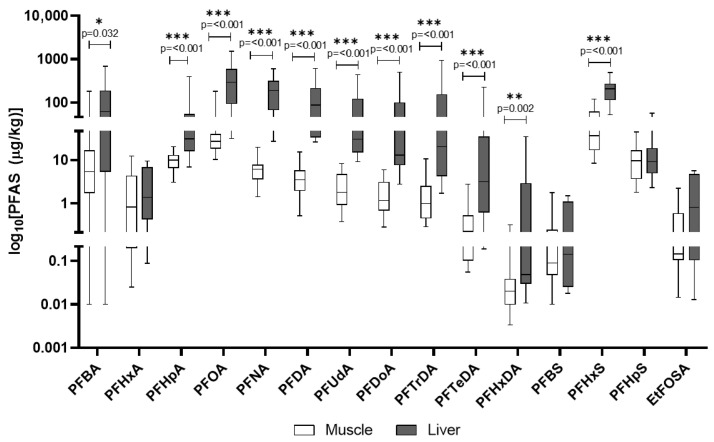
PFASs detected in wild boar muscle and liver. The *y*-axis is plotted on a log_10_ scale. Values are reported in µg/kg. Only compounds above the LOQ are shown.

In wild boar liver, PFOA, PFNA, and PFHxS were the most abundant (mean concentrations exceeding 200 μg/kg). Muscle tissue generally showed lower concentrations, though still relevant (Figure 7). Further PFASs that were part of the screening process and were detected, but unquantified, are presented in Table S8.

**Figure 8:**
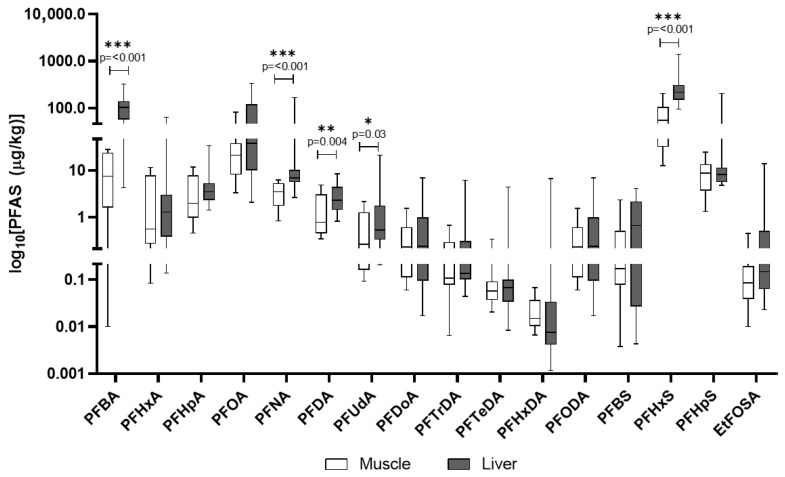
PFASs detected in domestic pigs’ muscle and liver. The *y*-axis is plotted on a log_10_ scale. Values are reported in µg/kg. Only compounds above the LOQ are shown.

In domestic pigs, PFHxS had the highest mean concentration in liver (311.5 μg/kg), followed by PFBA and PFOA (Table 6). Muscle levels were consistently lower than in liver, yet some compounds like PFHpS and EtFOSAA were present at meaningful concentrations (Figure 8). The other PFASs included in the analytical screening of domestic pigs’ tissues and found to be below quantification limits are detailed in Table S9.

**Figure 9:**
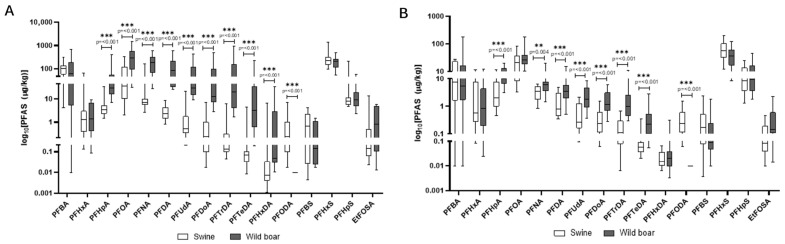
Comparison of concentrations of PFAS in the liver (**A**) and muscle (**B**) of wild boars and domestic pigs. The data are reported in μg/kg. Only compounds above the LOQ are shown.

In Figure 9A,B, the comparison of PFAS concentrations in the liver and muscle of wild boars and domestic pigs is reported. In wild boar, PFOA, PFNA, and PFHxS were the most abundant compounds in the liver, often reaching concentrations several-fold higher than in domestic pigs, suggesting species-specific retention or metabolic differences. Domestic pigs’ liver showed particularly high levels of PFHxS and PFBA, indicating possible exposure to different sources such as contaminated feed or water in agricultural settings. Despite lower overall PFAS concentrations, wild boar tissues displayed a broader range of detected compounds.

## 4. Discussion

This discussion focuses on the comparative analysis of PAHs, PCBs, and PFASs in wild boars and domestic pigs, emphasizing differences in tissue accumulation, exposure pathways, and regulatory relevance. The results of this study highlight significant interspecies and tissue-specific differences in the presence and distribution of PAH, PCB, and PFAS. These differences reflect distinct ecological exposures and feeding practices, offering new insights into bioaccumulation patterns and potential health risks.

PAHs are a large group of compounds with diverse toxicological profiles. While some do not cause severe adverse effects, others, like benzo[a]pyrene, are carcinogens and mutagens, a property that explains their regulation in food safety standards [31]. Their lipophilic nature means they accumulate in fatty tissues, but they are also metabolized by the liver, which explains the higher concentrations often seen in that organ [31]. In wild boars, a variety of PAHs were detected, with concentrations generally higher in the liver compared to the muscle tissue. Specifically, pyrene, fluoranthene, and anthracene exhibited the highest mean concentrations in the liver. Statistically significant differences (*p* < 0.001) were observed between liver and muscle for most compounds, indicating preferential accumulation in the liver. The detection frequency (DF) for individual compounds varied; for instance, pyrene was detected in 64.9% of samples, while anthracene was found in over 89% of liver samples. Compounds such as benzo[b,j]fluoranthene and benzo[k]fluoranthene were present at lower concentrations, with detection frequencies around 78%. The PAH levels detected in wild boar liver in this study were lower than those reported by González-Gómez et al. [32], where benzo[a]pyrene and other high molecular weight PAHs were present in significantly higher concentrations, indicating potential regional differences in contamination sources [32]. In domestic pigs, pyrene, anthracene, and fluoranthene were also prominent, with pyrene showing particularly high levels in the liver and a detection frequency of 81%. The observed high levels of certain PAHs in domestic pigs, particularly in the liver, align with findings from a study indicating that manufactured feed is a primary source of PAH intake for livestock [33]. Moreover, our findings on high pyrene and fluoranthene levels in domestic pigs’ liver are consistent with data from Ciganek et al. (2006), who attributed elevated PAHs in pig tissues to airborne contamination and feed exposure in farm settings [34].

Specific PAHs that are included within Regulation (EU) 2023/915 [35], specifically benzo[a]pyrene, benzo[a]anthracene, benzo[b]fluoranthene, and chrysene, are compounds for which Maximum Levels (MLs) in food have been established, particularly due to their potential to originate from environmental contamination due to natural phenomena such as fires or from anthropogenic activities. In the present study, these four PAHs were included among the target analytes; however, they were not detected in either tissue matrix or in either of the two species examined [35]. When comparing the two species, domestic pigs’ tissues generally exhibited markedly higher concentrations of most PAHs, especially in the liver, where pyrene and fluoranthene levels significantly exceeded those found in wild boar. In contrast, wild boar liver displayed a greater variety of detected PAHs, despite their generally lower individual concentrations. Anthracene was detected in both species with comparable frequencies, but its concentrations in domestic pigs’ muscle were significantly higher, pointing to possible differences in metabolic processing or source exposure [24]. When comparing species, it becomes evident that wild boars and domestic pigs differ not only in the concentration levels but also in the variety of PAHs accumulated, reflecting their contrasting diets and environmental exposures [36]. Unlike wild boar, domestic pigs’ showed a more even distribution of PAHs between muscle and liver, and some PAHs, such as phenanthrene, were detected in domestic pigs’ muscle but were absent or below quantification limits in wild boar. As opportunistic omnivores, wild boar engage in extensive foraging that involves digging for roots, tubers, and invertebrates in the soil. This direct interaction with diverse environmental matrices exposes them to a broad spectrum of soil-bound PAHs, which explains the greater variety of compounds found in their tissues [37]. For domestic pigs, manufactured feed is identified as the overwhelming primary source of PAH intake, accounting for approximately 99% of their total exposure. This concentrated dietary exposure, potentially from contaminated agricultural feedstuffs or specific feed processing practices, explains the significantly higher overall concentrations of certain PAHs observed in domestic pigs [38]. High-temperature food processing methods, such as smoking, grilling, and roasting, are known to substantially increase PAH content in meat products, and the fat content of meat also plays a role due to fat pyrolysis and the lipophilic nature of PAHs [39]. Overall, PAH contamination appears to be driven by species-specific behaviors: environmental foraging in wild boars leads to broader compound profiles, while controlled feeding in domestic pig results in higher concentrations of specific PAHs, primarily introduced via feed and farm practices.

In addition to PAHs, this study reveals distinct accumulation patterns of PCBs across tissues and species, which further emphasize the role of environmental exposure routes. PCBs are persistent, fat-soluble compounds that are highly resistant to degradation. This persistence and lipophilicity lead to their biomagnification [40]. The toxicity of PCBs varies by congener. Some, particularly dioxin-like PCBs (such as PCB 126), are highly toxic, acting similarly to dioxins by disrupting endocrine function and having immunotoxic and carcinogenic effects [40]. The stability of PCBs means they pose a long-term risk to both wildlife and humans consuming contaminated tissues. In the wild boar, muscle tissue exhibited a broader profile of detectable PCB congeners compared to the liver, where only a limited number were quantifiable. Key congeners such as PCB 44, 123, 156 were found at substantial concentrations in muscle, with high detection frequencies (e.g., detected in over 95% of muscle samples). The muscle tissue of wild boars emerges as a hotspot for PCBs’ bioaccumulation, possibly due to a combination of direct environmental exposure and the lipophilic nature of many congeners. Dioxin-like PCBs, including PCB 126 and PCB 77, were also predominantly detected in muscle tissue at higher frequencies and concentrations than in liver. PCB concentrations in wild boar muscle in this study exceed those reported by Tomza-Marciniak et al. (2014) in wild boars from Poland, highlighting a potentially greater exposure in our study area, likely due to differing land use and industrial activity [41]. Conversely, domestic pigs displayed a more evenly distributed pattern of PCB accumulation between liver and muscle tissues. PCB 180 and PCB 123 were consistently detected in muscle (100% and 89% detection frequency, respectively) and at measurable levels in liver (PCB 180). The PCB congener distribution observed in domestic pigs is similar to the findings of Hoogenboom et al. (2004), who reported elevated levels of indicator PCBs such as PCB 123 and 180 in pigs fed contaminated feed, underscoring feed as a primary exposure route [42]. The PCBs identified in this study include several relevant congeners. Specifically, PCB 28, PCB 52, PCB 153, and PCB 180 are among the six “indicator PCBs” (ICE PCBs) [35], which also include PCB 101 and PCB 138, used as markers for the presence of non-dioxin-like PCBs (NDL-PCBs) in food and feed by the European Union. Furthermore, several detected congeners, such as PCB 77, PCB 81, PCB 126, PCB 156, PCB 157, and PCB 167, are classified as dioxin-like PCBs due to their toxicological properties [35]. EU legislation, including recent amendments effective from January 2023, sets MLs for dioxins and PCBs in various foodstuffs, including wild boar meat, to protect public health. The presence of both indicator PCBs and dioxin-like PCBs in animal tissues raises concerns, not only for wildlife health but also for human consumption of game and livestock products [35]. A direct comparison revealed that overall PCB levels were generally higher in wild boar muscle than in domestic pigs, with several congeners (e.g., PCB 28, PCB 31) showing mean concentrations more than double in wild animals. The diversity of PCB congeners was also greater in wild boar muscle, reflecting their varied and uncontrolled environmental exposure through foraging in diverse habitats [43]. In contrast, domestic pigs, with their typically controlled diets, likely accumulate PCBs primarily through contaminated feed, leading to a different congener profile and tissue distribution [42]. Dioxin-like PCBs were found at higher frequencies and concentrations in wild boar muscle compared to liver, whereas in domestic pigs, these levels were generally lower and more evenly distributed. This underscores the influence of lifestyle, diet, and habitat on contaminant exposure and subsequent bioaccumulation [44].

PFASs are unique due to their extreme stability and a combination of lipo- and hydrophilic properties, which allows them to bind to proteins in the blood and organs rather than just fat [13]. This characteristic results in their accumulation primarily in organs like the liver and kidneys, as observed in our study. PFASs are known to be endocrine disruptors and have been linked to a wide range of health issues, including liver damage, immune system effects, and developmental problems. Their persistence in the environment and in biological systems, combined with their mobility in water, makes them a major public health concern [13]. All analyzed PFASs were detected in 100% of wild boar liver and muscle samples, indicating widespread environmental contamination. Liver tissues consistently showed higher concentrations than muscle. The most predominant PFASs in liver were Perfluorooctanoic acid (PFOA), Perfluorononanoic acid (PFNA), and Perfluorohexane sulfonic acid (PFHxS), with mean concentrations often exceeding 200 µg/kg. The exceptionally high PFOA and PFNA concentrations in wild boar liver align with findings by Death et al. (2021), who reported wild game species can accumulate substantial PFAS burdens, particularly in regions affected by industrial or landfill runoff [20]. Similarly, all analyzed PFASs were detected in 100% of domestic pig liver and muscle samples, highlighting contamination within agricultural settings. PFAS contamination was evaluated in light of EU Regulation 2023/915, which defines maximum allowable levels in meat and offal, making this assessment particularly relevant for food safety evaluations Liver tissues also showed higher PFAS concentrations than muscle. In domestic pigs, PFHxS was most abundant in liver, followed by PFBA and PFOA. The presence of PFASs in domestic pigs suggests contamination pathways within agricultural production, such as contaminated feed or water or the use of PFAS-polluted fertilizers like sewage sludge on feed crops [20,45]. Similar PFHxS and PFBA’s dominance in domestic pigs’ liver was documented by Mikołajczyk et al. (2023) in dairy animals, supporting the idea that agricultural exposure via water or contaminated feed is a significant PFAS pathway in livestock [19]. The EU regulates four key PFAS compounds (PFOS, PFOA, PFNA, PFHxS) and their sum in food, based on a group tolerable weekly intake (TWI) of 4.4 ng/kg body weight per week. Commission Regulation (EU) 2023/915 [35] sets maximum levels for these PFASs in various food categories [35]. A direct comparison reveals significant non-compliance. Mean concentrations of PFOA, PFNA, and PFHxS in wild boar liver and muscle are profoundly above EU maximum levels for game offal and meat. Similarly, mean concentrations in domestic pig liver and muscle are substantially higher than EU limits for pig offal and meat [35].

The analysis of PFAS profiles and accumulation patterns between wild boar and domestic pigs reveals distinct differences, primarily attributable to their divergent ecological niches and feeding behaviors and the resulting exposure pathways. While both species exhibited ubiquitous PFAS contamination across all analyzed tissues, the specific compounds dominating their profiles and their respective concentrations varied considerably. A notable observation was the significantly higher concentrations of long-chain PFASs, such as PFOA and PFNA, in wild boar liver compared to domestic pigs, often by several-fold (i.e., PFOA). The broader spectrum of detected PFASs in wild boar tissues, particularly the liver, further supports the notion of a more varied exposure landscape for these wild populations [46]. In contrast, domestic pigs, despite also showing 100% detection rates for PFASs, exhibited a different dominant profile, with particularly high levels of PFHxS and PFBA in their liver tissues. This pattern points towards more specific, potentially localized, and ongoing contamination sources within agricultural production systems [47]. Although both species exhibited universal PFAS detection, the compound-specific profiles suggest different contamination pathways, which are predominantly a diffuse environmental exposure for wild boars and a point-source agricultural contamination for domestic pigs. Unlike wild animals, domestic pigs are typically raised in controlled environments with managed diets [24]. Their exposure to PFASs is therefore largely mediated through their feed and drinking water. This aligns with established knowledge that livestock can be exposed to PFASs through contaminated feedstuffs and water. A particularly concerning pathway is the use of PFAS-polluted fertilizers, such as sewage sludge, which can contaminate crops used for animal feed [48].

## 5. Conclusions

This study highlights significant differences in contaminant bioaccumulation between wild boars and domestic pigs, influenced by their distinct exposure routes, feeding behaviors, and environments. Wild boars showed a broader range of contaminants, particularly PCBs in muscle and PFASs in liver, reflecting diffuse environmental exposure. In contrast, domestic pigs had much higher concentrations, especially of PAHs, primarily due to contaminated feed in controlled farming settings. The liver’s central role in toxicant retention was consistent across both species for all three contaminant classes. A key concern is that concentrations of several regulated PFASs in both wild and farmed animals exceeded EU maximum levels (sum of PFOS, PFOA, PFNA, and PFHxS: 1.3 µg/kg), raising immediate food safety concerns. Livestock serves as a direct route of human exposure to contaminants, emphasizing the critical need for thorough monitoring. Emerging contaminants like PFASs are particularly alarming, as even low doses can disrupt hormonal regulation, metabolism, growth, and immune function. Their presence in food-producing animals poses risks to both animal welfare and human health.

These findings underscore the need for environmental biomonitoring programs to account for species- and tissue-specific differences in contaminants’ accumulation and regional contamination patterns. This research provides crucial data for updating toxicological thresholds and dietary intake guidelines. Continuous environmental monitoring, stricter control of agricultural contaminant sources, and updated risk assessments for both wild and domestic meat products are essential to protect animal welfare and human health. Further research should investigate long-term health implications, co-exposure effects, and the effectiveness of current mitigation strategies. Ultimately, this interdisciplinary approach is vital for integrating ecotoxicological data into public health frameworks, using sentinel species for early detection, and ensuring standardized methodologies to address the complex challenges of persistent and emerging contaminants for the well-being of ecosystems, animals, and human populations.

## Figures and Tables

**Table 1:** Summary statistics (mean ± SD, percentiles, min–max) and detection frequency (DF%; percentage of samples for which compounds were quantified above the respective LOD) for PAHs quantified in wild boar tissues. Values in μg/kg.

				Percentile				
	Matrix	Mean ± SD	Min–Max	25th	Median	75th	*p*-Value	DF (%)
Naphthalene	Muscle	0.084 ± 0.049	0–0.13	0.075	0.11	0.12	<0.001	76.9
	Liver	1.139 ± 0.625	0–2.03	0.99	1.35	1.47		81.1
Acenaphthylene	Muscle	N.D.					<0.001	0
	Liver	0.232 ± 0.125	0–0.5	0.21	0.26	0.29		83.8
Acenaphthene	Muscle	0.055 ± 0.015	0.01–0.08	0.05	0.05	0.07	<0.001	100
	Liver	N.D.						0
Fluorene	Muscle	N.D.					<0.001	0
	Liver	0.327 ± 0.145	0–0.73	0.31	0.35	0.4		89.2
Anthracene	Muscle	0.248 ± 0.079	0–0.33	0.24	0.27	0.29	<0.001	92.3
	Liver	1.813 ± 0.914	0–5.32	1.59	1.8	2.11		89.2
Pyrene	Muscle	N.D.					<0.001	0
	Liver	3.826 ± 2.934	0–7.6	0	5.57	6.19		64.9
Fluoranthene	Muscle	0.293 ± 0.145	0–0.59	0.215	0.25	0.435	<0.001	94.9
	Liver	3.653 ± 1.369	0–7.44	3.36	3.8	4.25		94.6
Benzo_e_pyrene	Muscle	N.D.					<0.001	0
	Liver	0.674 ± 0.393	0–1.16	0.67	0.85	0.9		78.4
Benzo_k_fluoranthene	Muscle	N.D.					<0.001	0
	Liver	0.047 ± 0.025	0–0.09	0.04	0.05	0.06		81.1
Benzo_b,j_fluoranthene	Muscle	N.D.					<0.001	0
	Liver	0.023 ± 0.014	0–0.04	0.01	0.03	0.03		78.4

**Table 2:** Summary statistics (mean ± SD, percentiles, min–max) and detection frequency (DF%; percentage of samples for which compounds were quantified above the respective LOD) for PAHs in domestic pigs’ tissues. Values in μg/kg.

				Percentile				
	Matrix	Mean ± SD	Min–Max	25th	Median	75th	*p*-Value	DF (%)
Naphthalene	Muscle	0.758 ± 0.457	0–2.15	0.665	0.76	0.865	<0.001	89.5
	Liver	3.49 ± 3.085	0–18.43	2.59	3.28	4.18		83.8
Acenaphthylene	Muscle	0.076 ± 0.346	0–2.14	0.01	0.01	0.01	<0.001	84.2
	Liver	0.166 ± 0.101	0–0.49	0.15	0.16	0.193		86.5
Acenaphthene	Muscle	0.006 ± 0.039	0–0.24	0	0	0	0.337	2.63
	Liver	N.D.						0
Fluorene	Muscle	0.124 ± 0.290	0–1.41	0.05	0.06	0.07	<0.001	86.8
	Liver	0.279 ± 0.167	0–0.62	0.24	0.28	0.38		81.1
Anthracene	Muscle	0.867 ± 0.531	0–2.25	0.36	1.105	1.178	<0.001	84.2
	Liver	1.641 ± 0.972	0–3.39	0.65	2.01	2.19		83.8
Phenanthrene	Muscle	0.01 ± 0.046	0–0.25	0	0	0	0.166	5.3
	Liver	N.D.						0.000
Pyrene	Muscle	1.075 ± 0.831	0–4.25	0.983	1.08	1.228	<0.001	81.6
	Liver	9.294 ± 7.048	0–35.31	5.92	9.35	11.29		81.1
Fluoranthene	Muscle	0.159 ± 0.321	0–1.36	0.063	0.08	0.08	<0.001	76.4
	Liver	7.361 ± 5.406	0–22.32	4.8	7.39	8.81		81.1
Benzo_e_pyrene	Muscle	0.009 ± 0.032	0–0.14	0	0	0	<0.001	7.9
	Liver	0.866 ± 0.595	0–2.71	0.71	0.92	1.15		81.1

**Table 3:** Summary statistics (mean ± SD, percentiles, min–max) and detection frequency (DF%; percentage of samples for which compounds were quantified above the respective LOD) for PCBs in liver and muscle of wild boar. Values are reported in μg/kg.

				Percentile				
	Matrix	Mean ± SD	Min–Max	25th	Median	75th	*p*-Value	DF%
PCB 31	Liver	4.045 ± 3.008	0–7.867	0	5.533	6.203	<0.001	66.7
	Muscle	11.689 ± 7.088	0–30.818	7.951	12.622	14.238		70.940
PCB 28	Liver	N.D.					<0.001	0
	Muscle	15.943 ± 11.619	0–45.614	8.406	17.722	19.615		76.9
PCB 18	Liver	0.037 ± 0.018	0–0.056	0.034	0.046	0.050	<0.001	82.1
	Muscle	11.504 ± 6.673	0–27.826	9.926	12.63	14.478		82.1
PCB 81	Liver	N.D.					<0.001	0
	Muscle	2.078 ± 1.222	0–6.156	1.606	2.062	2.417		87.2
PCB 77	Liver	0.235 ± 0.094	0–0.314	0.243	0.265	0.285	<0.001	87.2
	Muscle	0.836 ± 0.483	0–2.291	0.637	0.809	0.963		92.3
PCB 99	Liver	N.D.					<0.001	0
	Muscle	0.668 ± 0.382	0–1.576	0.505	0.643	0.787		89.7
PCB 95	Liver	N.D.					<0.001	0
	Muscle	0.188 ± 0.081	0–0.499	0.165	0.179	0.189		97.4
PCB 126	Liver	0.205 ± 0.094	0–0.299	0.193	0.231	0.266	<0.001	84.6
	Muscle	0.395 ± 0.247	0–1.169	0.309	0.383	0.466		89.7
PCB 52	Liver	N.D.					<0.001	0
	Muscle	0.148 ± 0.068	0–0.366	0.131	0.144	0.168		92.3
PCB 123	Liver	N.D.					<0.001	0
	Muscle	0.154 ± 0.089	0–0.419	0.113	0.146	0.178		92.3
PCB 44	Liver	N.D.					<0.001	0
	Muscle	0.376 ± 0.199	0–0.980	0.300	0.3576	0.406		97.4
PCB 167	Liver	N.D.					<0.001	0
	Muscle	0.137 ± 0.075	0–0.361	0.113	0.137	0.151		97.4
PCB 157	Liver	N.D.					<0.001	0
	Muscle	0.442 ± 0.217	0–1.075	0.390	0.452	0.513		94.9
PCB 156	Liver	N.D.					<0.001	0
	Muscle	0.651 ± 0.222	0.224–1.426	0.575	0.635	0.685		100
PCB 153	Liver	N.D.					<0.001	0
	Muscle	0.167 ± 0.099	0–0.502	0.134	0.163	0.190		89.7
PCB 187	Liver	N.D.					<0.001	0
	Muscle	1.669 ± 1.117	0–4.024	1.034	1.756	2.110		82.1
PCB 180	Liver	0.224 ± 0.252	0–0.669	0	0	0.438	0.316	46.2
	Muscle	0.312 ± 0.201	0–0.907	0.232	0.3	0.368		87.2
PCB 170	Liver	N.D.					<0.001	0
	Muscle	1.973 ± 1.390	0–6.242	1.498	2.209	2.416		76.9

**Table 4:** Summary statistics (mean ± SD, percentiles, min–max) and detection frequency (DF%; percentage of samples for which compounds were quantified above the respective LOD) for PCBs in liver and muscle of domestic pigs. Values are reported in μg/kg.

				Percentile				
	Matrix	Mean ± SD	Min–Max	25th	Median	75th	*p*-Value	DF%
PCB 31	Muscle	2.696 ± 1.265	0–5.67	1.915	2.84	3.675	<0.001	92.3
	Liver	0.52 ± 0.315	0–1.02	0.28	0.495	0.828		94.7
PCB 28	Muscle	5.896 ± 4.169	0–18.15	4.69	6.1	7.545	<0.001	76.9
	Liver	N.D.						0
PCB 18	Muscle	3.971 ± 1.114	0–7.67	3.57	3.97	4.275	<0.001	97.4
	Liver	0.048 ± 0.009	0–0.06	0.05	0.05	0.05		97.4
PCB 81	Muscle	1.529 ± 1.011	0–4.5	1.125	1.63	1.935	<0.001	82.1
	Liver	N.D.						0
PCB 77	Muscle	0.7903 ± 0.349	0–1.43	0.71	0.8	0.98	<0.001	89.7
	Liver	0.233 ± 0.106	0–0.32	0.225	0.28	0.29		84.2
PCB 99	Muscle	0.594 ± 0.351	0–1.64	0.45	0.63	0.8	<0.001	84.6
	Liver	N.D.						0
PCB 95	Muscle	0.089 ± 0.052	0–0.28	0.07	0.09	0.11	<0.001	89.7
	Liver	N.D.						0
PCB 126	Muscle	0.238 ± 0.172	0–0.9	0.14	0.23	0.31	0.943	84.6
	Liver	0.219 ± 0.068	0–0.28	0.2225	0.24	0.25		92.1
PCB 52	Muscle	0.104 ± 0.043	0–0.26	0.09	0.11	0.12	<0.001	92.3
	Liver	N.D.						0
PCB 123	Muscle	0.102 ± 0.051	0.01–0.27	0.085	0.1	0.12	<0.001	89.7
	Liver	N.D.						0
PCB 44	Muscle	0.331 ± 0.156	0–0.84	0.275	0.32	0.4	<0.001	92.3
	Liver	N.D.						0
PCB 167	Muscle	0.089 ± 0.038	0–0.16	0.08	0.09	0.1	<0.001	94.9
	Liver	N.D.						0
PCB 157	Muscle	0.033 ± 0.014	0–0.07	0.03	0.03	0.04	<0.001	92.3
	Liver	N.D.						0
PCB 156	Muscle	0.531 ± 0.307	0–1.49	0.41	0.52	0.61	<0.001	97.4
	Liver	N.D.						0
PCB 187	Muscle	1.128 ± 0.430	0–2.23	0.95	1.17	1.32	<0.001	97.4
	Liver	0.329 ± 0.459	0–1.33	0	0	0.87		36.8
PCB 180	Muscle	0.199 ± 0.077	0.02–0.42	0.16	0.19	0.215	0.232	100
	Liver	0.130 ± 0.108	0–0.26	0	0.2	0.21		60.5
PCB 170	Muscle	1.697 ± 1.151	0–4.42	1.21	1.83	2.175	<0.001	79.5
	Liver	0.2295 ± 0.598	0–1.9	0	0	0		13.2

**Table 5:** Summary statistics (mean ± SD, percentiles, min–max) and detection frequency (DF%; percentage of samples for which compounds were quantified above the respective LOD) of PFASs in liver and muscle of wild boar. Values are reported in μg/kg.

				Percentile				
	Matrix	Mean ± SD	Min–Max	25th	Median	75th	*p*-Value	DF (%)
PFBA	liver	136.247 ± 189.297	0.01–680.282	7.000	61.226	185.1313	0.032	100
	muscle	23.608 ± 49.374	0.01–179.662	1.976	5.351	12.297		100
PFHxA	liver	3.191 ± 3.308	0.088–9.377	0.503	1.372	6.240	0.493	100
	muscle	2.607 ± 3.812	0.024–12.301	0.234	0.826	4.186		100
PFHpA	liver	56.783 ± 99.729	6.9113–397.112	17.970	30.509	48.939	<0.001	100
	muscle	10.130 ± 4.775	3.054–20.362	6.730	9.999	12.712		100
PFOA	liver	424.370 ± 438.077	31.335–1504.985	99.445	292.4903	545.4161	<0.001	100
	muscle	42.650 ± 44.605	10.323–180.455	18.757	26.799	37.835		100
PFNA	liver	208.616 ± 166.745	26.989–593.141	71.521	189.262	301.8965	<0.001	100
	muscle	6.909 ± 4.753	1.414–19.4518	4.690	6.098	7.289		100
PFDA	liver	157.963 ± 177.841	26.072–603.357	35.768	86.622	201.336	<0.001	100
	muscle	4.277 ± 3.57	0.518–15.288	2.359	3.518	5.094		100
PFUdA	liver	96.083 ± 136.718	9.103–437.891	17.819	30.186	108.88	<0.001	100
	muscle	2.9552 ± 2.3916	0.378–8.330	1.057	1.797	4.350		100
PFDoA	liver	84.858 ± 149.844	2.774–498.632	9.163	12.938	85.087	<0.001	100
	muscle	1.961 ± 1.731	0.284–5.921	0.844	1.166	2.743		100
PFTrDA	liver	154.306 ± 292.098	1.715–926.597	4.991	20.271	132.8584	<0.001	100
	muscle	2.056 ± 2.626	0.290–10.611	0.573	0.985	2.134		100
PFTeDA	liver	34.794 ± 69.701	0.187–225.411	0.642	3.181	28.455	<0.001	100
	muscle	0.484 ± 0.686	0.055–2.784	0.104	0.228	0.509		100
PFHxDA	liver	4.433 ± 9.954	0.010–34.644	0.033	0.049	2.478	0.002	100
	muscle	0.041 ± 0.074	0.003–0.318	0.01	0.02	0.026		100
PFBS	liver	0.440 ± 0.551	0.017–1.505	0.039	0.142	0.883	0.625	100
	muscle	0.284 ± 0.464	0.01–1.772	0.061	0.090	0.176		100
PFHxS	liver	208.090 ± 110.686	52.491–485.835	123.773	207.958	259.3407	<0.001	100
	muscle	42.062 ± 29.379	8.476–120.058	17.640	36.433	56.497		100
PFHpS	liver	15.221 ± 16.663	2.319–56.727	5.288	9.091	12.187	0.769	100
	muscle	12.309 ± 11.609	1.796–44.284	3.937	9.597	15.365		100
EtFOSA	liver	2.109 ± 2.278	0.012–5.678	0.154	0.809	4.527	0.077	100
	muscle	0.406 ± 0.577	0.014–2.214	0.110	0.144	0.307		100

**Table 6:** Summary statistics (mean ± SD, percentiles, min–max) and detection frequency (DF%; percentage of samples for which compounds were quantified above the respective LOD) of PFASs in liver and muscle of domestic pigs. Values are reported in μg/kg.

				Percentile				
	Matrix	Mean	Min–Max	25th	Median	75th	*p*-Value	DF (%)
PFBA	liver	115.214 ± 86.849	4.256–322.080	72.370	103.246	138.467	<0.001	100
	muscle	11.314 ± 10.448	0.01–27.924	1.778	7.482	23.065		100
PFHxA	liver	6.518 ± 16.320	0.138–63.430	0.543	1.302	2.492	0.244	100
	muscle	2.963 ± 4.234	0.084–11.516	0.291	0.563	5.847		100
PFHpA	liver	5.891 ± 7.989	1.438–33.477	2.592	3.488	4.926	0.108	100
	muscle	3.921 ± 3.948	0.462–11.698	1.064	1.982	6.979		100
PFOA	liver	78.211 ± 94.251	2.086–335.428	16.317	37.301	104.700	0.108	100
	muscle	24.427 ± 19.836	3.314–81.040	9.314	20.910	35.396		100
PFNA	liver	18.655 ± 41.295	2.618–166.665	5.846	6.910	9.033	<0.001	100
	muscle	3.406 ± 1.815	0.838–6.262	1.976	3.474	5.187		100
PFDA	liver	3.331 ± 2.41	0.832–8.414	1.468	2.305	4.266	0.004	100
	muscle	1.489 ± 1.416	0.349–4.831	0.481	0.792	2.577		100
PFUdA	liver	2.629 ± 5.331	0.206–20.933	0.347	0.534	1.509	0.03	100
	muscle	0.642 ± 0.698	0.093–2.163	0.171	0.265	1.008		100
PFDoA	liver	1.291 ± 2.085	0.017–6.927	0.126	0.240	0.908	0.63	100
	muscle	0.426 ± 0.453	0.059–1.551	0.122	0.235	0.512		100
PFTrDA	liver	0.993 ± 1.849	0.044–6.166	0.110	0.135	0.290	0.361	100
	muscle	0.189 ± 0.174	0.006–0.671	0.083	0.108	0.288		100
PFTeDA	liver	0.483 ± 1.170	0.008–4.406	0.037	0.068	0.092	0.682	100
	muscle	0.087 ± 0.093	0.021–0.341	0.041	0.057	0.085		100
PFHxDA	liver	0.479 ± 1.719	0.001–6.687	0.005	0.008	0.025	0.126	100
	muscle	0.025 ± 0.019	0.006–0.067	0.011	0.015	0.032		100
PFODA	liver	1.291 ± 2.085	0.017–6.927	0.126	0.240	0.908	0.63	100
	muscle	0.426 ± 0.453	0.059–1.551	0.122	0.235	0.512		100
PFBS	liver	1.093 ± 1.219	0.004–4.147	0.031	0.661	1.912	0.274	100
	muscle	0.392 ± 0.587	0.004–2.334	0.081	0.172	0.327		100
PFHxS	liver	311.489 ± 315.703	95.208–1401.28	156.254	217.754	303.530	<0.001	100
	muscle	76.412 ± 58.136	12.447–201.525	34.012	55.132	99.195		100
PFHpS	liver	27.155 ± 54.162	4.777–202.771	5.825	8.176	11.197	0.656	100
	muscle	9.40 ± 6.743	1.334–24.235	3.937	8.633	12.841		100
EtFOSAA	liver	1.254 ± 3.495	0.023–13.743	0.069	0.146	0.459	0.259	100
	muscle	0.139 ± 0.131	0.01–0.455	0.050	0.086	0.156		100

## Data Availability

The original contributions presented in this study are included in the article and Supplementary Material. Further inquiries can be directed to the corresponding author.

## Supplementary Materials

The following supporting information can be downloaded at: https://www.mdpi.com/article/10.3390/ani15172600/s1, Table S1. The table shows the validation parameters calculated for the analyzed PCB and PAH compounds: LOD, LOQ, linearity range, and recovery; Table S2. The table shows the analyzed PCDD and PCDF compounds; Table S3. The table shows the validation parameters calculated for the analyzed dioxin compounds: LOD, LOQ, linearity range, and recovery; Table S4. The table shows the analyzed PFAS compounds; Table S5. Linearity, LOD, and LOQ for PFASs; Table S6. Summary statistics (mean ± SD, percentiles, min–max) for target PAH in wild boar tissues. Values in μg/kg; Table S7. Summary statistics (mean ± SD, percentiles, min–max) for target PAH in wild boar tissues. Values in μg/kg; Table S8. Mean ± SD, median with percentile, and ranges of target PCB in liver and muscle of wild boar. Values are reported in μg/kg; Table S9. Mean ± SD, median with percentile, and ranges of target PCB in liver and muscle of domestic pigs. Values are reported in μg/kg; Table S10. Mean ± SD, median with percentile, and ranges of target PFAS in liver and muscle of wild boar. Values are reported in μg/kg; Table S11. Mean ± SD, median with percentile, and ranges of target PFAS in liver and muscle of domestic pigs. Values are reported in μg/kg.
