# Supplementary material for: Environmental Monitoring of PAHs, PCBs, PCDDs, PCDFs, and PFASs in Wild Boar and Domestic Pig Tissues from Northern Italy
Source: Animals (Basel). 2025 Sep 4;15(17):2600. doi: 10.3390/ani15172600 (PMC12427392; doi:10.3390/ani15172600)
Supplement: Supplementary file 1 [file animals-15-02600-s001.zip › animals-3819821-supplementary.pdf]

SUPPLEMENTARY INFORMATIONS

Table S1. The table shows the validation parameters calculated for the analyzed PCB and PAH compounds: LOD, LOQ, linearity range, and recovery.

PCB	Linea Range ug/L	R2	LOD	LOQ
**epta_pcb183**	0,001-50	0.9982	0.0012	0.0036
**epta_pcb177**	0,001-50	0.9993	0.0014	0.0042
**epta_pcb187**	0,001-50	0.9975	0.0015	0.0045
**epta_pcb189**	0,001-50	0.9987	0.0011	0.0033
**epta_pcb180**	0,001-50	0.9983	0.0012	0.0036
**epta_pcb170**	0,001-50	0.9948	0.0013	0.0039
**epta_pcb183**	0,001-50	0.9987	0.0012	0.0036
**epta_pcb187**	0,001-50	0.9989	0.001	0.003
**epta_pcb177**	0,001-50	0.9987	0.0014	0.0042
**epta_pcb170**	0,001-50	0.9998	0.0014	0.0042
**epta_pcb189**	0,001-50	0.9958	0.0013	0.0039
**epta_pcb180**	0,001-50	0.9964	0.0015	0.0045
**esa_pcb167**	0,001-50	0.9997	0.0012	0.0036
**esa_pcb157**	0,001-50	0.9988	0.001	0.003
**esa_pcb138**	0,001-50	0.9994	0.0012	0.0036
**esa_pcb169**	0,001-50	0.9984	0.0013	0.0039
**esa_pcb156**	0,001-50	0.9985	0.0011	0.0033
**esa_pcb149**	0,001-50	0.9987	0.0013	0.0039
**esa_pcb146**	0,001-50	0.9968	0.0014	0.0042
**esa_pcb128**	0,001-50	0.9982	0.0012	0.0036
**esa_pcb153**	0,001-50	0.9983	0.001	0.003
**esa_pcb151**	0,001-50	0.9974	0.0012	0.0036
**esa_pcb138**	0,001-50	0.9968	0.0013	0.0039
**esa_pcb167**	0,001-50	0.9983	0.0014	0.0042
**esa_pcb153**	0,001-50	0.9982	0.0015	0.0045

**esa_pcb151**	0,001-50	0.9979	0.0011	0.0033
**esa_pcb128**	0,001-50	0.9991	0.0014	0.0042
**esa_pcb156**	0,001-50	0.9993	0.0012	0.0036
**esa_pcb149**	0,001-50	0.9986	0.0013	0.0039
**esa_pcb146**	0,001-50	0.9974	0.001	0.003
**esa_pcb157**	0,001-50	0.9981	0.0012	0.0036
**esa_pcb169**	0,001-50	0.9989	0.0014	0.0042
**penta_pcb101**	0,001-50	0.9998	0.0012	0.0036
**penta_pcb95**	0,001-50	0.9978	0.0013	0.0039
**penta_pcb126**	0,001-50	0.9994	0.0012	0.0036
**penta_pcb123**	0,001-50	0.9996	0.0012	0.0036
**penta_pcb105**	0,001-50	0.9996	0.0014	0.0042
**penta_pcb118**	0,001-50	0.9989	0.0015	0.0045
**penta_pcb110**	0,001-50	0.9996	0.001	0.003
**penta_pcb99**	0,001-50	0.9987	0.0011	0.0033
**penta_pcb114**	0,001-50	0.9989	0.0011	0.0033
**penta_pcb110**	0,001-50	0.9986	0.0012	0.0036
**penta_pcb105**	0,001-50	0.9978	0.0014	0.0042
**penta_pcb99**	0,001-50	0.9985	0.0013	0.0039
**penta_pcb126**	0,001-50	0.9979	0.0011	0.0033
**penta_pcb114**	0,001-50	0.9965	0.0012	0.0036
**penta_pcb101**	0,001-50	0.9972	0.0012	0.0036
**penta_pcb123**	0,001-50	0.9968	0.0014	0.0042
**penta_pcb118**	0,001-50	0.9982	0.0011	0.0033
**penta_pcb95**	0,001-50	0.9991	0.0013	0.0039
**tetra_pcb81**	0,001-50	0.9964	0.0012	0.0036
**tetra_pcb77**	0,001-50	0.9967	0.0014	0.0042
**tetra_pcb52**	0,001-50	0.9977	0.0012	0.0036
**tetra_pcb44**	0,001-50	0.9968	0.001	0.003

**tetra_pcb81**	0,001-50	0.9994	0.0013	0.0039
**tetra_pcb52**	0,001-50	0.9985	0.0012	0.0036
**tetra_pcb77**	0,001-50	0.9986	0.0013	0.0039
**tetra_pcb44**	0,001-50	0.9996	0.0014	0.0042
**tri_pcb31**	0,001-50	0.999	0.0012	0.0036
**tri_pcb28**	0,001-50	0.999	0.0014	0.0042
**tri_pcb18**	0,001-50	0.9992	0.0013	0.0039
**tri_pcb31**	0,001-50	0.9998	0.0012	0.0036
**tri_pcb18**	0,001-50	0.9987	0.0011	0.0033
**tri_pcb28**	0,001-50	0.9988	0.0014	0.0042
**PAH**	**Linea Range ug/L**	**R2**	**LOD**	**LOQ**
**naphthalene**	0,001-50	0.9907	0.00124	0.00372
**acenaphthylene**	0,001-50	0.9918	0.0012	0.00366
**acenaphthene**	0,001-50	0.99	0.00121	0.00363
**fluorene**	0,001-50	0.9912	0.00123	0.00369
**anthracene**	0,001-50	0.9908	0.00125	0.00375
**phenanthrene**	0,001-50	0.9975	0.00127	0.00381
**pyrene**	0,001-50	0.9912	0.00114	0.00342
**fluoranthene**	0,001-50	0.9914	0.00163	0.00489
**benzo_a_anthracene**	0,001-50	0.9912	0.00187	0.00561
**crisene**	0,001-50	0.9923	0.00121	0.00363
**benzo_a_ pyrene**	0,001-50	0.9957	0.00118	0.00354
**perylene**	0,001-50	0.9963	0.00126	0.00378
**benzo_e_ pyrene**	0,001-50	0.9922	0.00124	0.00372
**benzo_k_fluoranthene**	0,001-50	0.9913	0.00118	0.00354
**benzo_b,j_fluoranthene**	0,001-50	0.9919	0.00121	0.00363
**dibenzo_a,e_pyrene**	0,001-50	0.9909	0.00127	0.00381
**dibenzo_ah_pyrene**	0,001-50	0.991	0.00124	0.00372
**benzo_ghi_perylene**	0,001-50	0.9912	0.00122	0.00366
**indeno 1,2,3 cd pyrene**	0,001-50	0.9893	0.00116	0.00348
**dibenzo_a,i_pyrene**	0,001-50	0.9907	0.00118	0.00354
**dibenzo_a,l_pyrene**	0,001-50	0.9908	0.00123	0.00369
**dibenzo_ah_anthracene**	0,001-50	0.9957	0.00124	0.00372

Table S2. The table shows the analyzed PCDDs and PCDFs compounds.

**rt**	**analite**	**precursor**	**product**	**Dwell**	**collision energy**
30	TCDD	321,9	258,9	75	24
	TCDD	319,9	256,9	75	24

	TCDF	305,9	242,9	75	33
	TCDF	303,9	240,9	75	33
47	PCDD	355,9	292,9	75	25
	PCDD	353,9	290,9	75	25
	PCDF	339,9	276,9	75	35
	PCDF	337,9	274,9	75	35
53	HxCDD	391,8	328,8	75	25
	HxCDD	389,8	326,9	75	25
	HxCDF	375,8	312,9	75	35
	HxCDF	373,8	310,9	75	35
56,5	HpCDD	425,8	362,8	75	25
	HpCDD	423,8	360,8	75	25
	HpCDF	409,8	346,8	75	36
	HpCDF	407,8	344,8	75	36
59,5	OCDD	459,7	396,8	75	26
	OCDD	457,7	394,8	75	26
	OCDF	443,7	380,8	75	35
	OCDF	441,7	378,8	75	35

Table S3. The table shows the validation parameters calculated for the analyzed Dioxin compounds: LOD, LOQ, linearity range, and recovery.

**Dioxin**	**Linea Range ug/L**	**R2**	**LOD**	**LOQ**
Dioxin TCDD	0,001-50	0.9912	0.001	0.003

Table S4. The table shows the analyzed PFASs compounds.

Compound Name	Precursor Ion m/z	Product Ion m/z	declustering potential	Collision Energy eV	Polarity
PFBA	212.9	169	-25	-12	negative
PFPeA	262.9	219	-20	-12	negative
PFHxA	313	269	-25	-12	negative
PFHpA	363	319	-25	-12	negative
PFOA	413	369	-25	-14	negative
PFNA	463	419	-25	-14	negative
PFDA	513	469	-25	-16	negative
PFUdA	563	519	-25	-18	negative
PFDoA	613	569	-25	-18	negative
PFTrDA	663	619	-25	-20	negative
PFTeDA	713	669	-25	-22	negative
PFHxDA	813	769	-25	-24	negative
PFODA	913	869	-25	-26	negative
PFBS	298.9	80	-55	-58	negative
PFHxS	399	80	-60	-74	negative
PFHpS	449	80	-65	-88	negative
PFOS	499	80	-65	-108	negative
PFDS	599	80	-85	-118	negative
PFOSA	498	78	-60	-85	negative
MeFOSA	512	169	-75	-37	negative
EtFOSA	526	169	-75	-37	negative
N-MeFOSAA	570	419	-40	-36	negative
N-EtFOSAA	584	419	-50	-36	negative
13C4_PFBA	217	172	-25	-12	negative
13C5_PFPeA	268	223	-20	-12	negative
13C2_PFHxA	315	270	-25	-12	negative
13C4_PFHpA	367	322	-25	-12	negative
13C4_PFOA	417	372	-25	-14	negative
13C5_PFNA	468	423	-25	-14	negative
13C2_PFDA	515	470	-25	-16	negative
13C2_PFUdA	565	520	-25	-18	negative
13C2_PFDoA	615	570	-25	-18	negative
13C4_PFOS	503	80	-65	-108	negative
d3MeFOSA	515	169	-75	-37	negative

Analytical Parameters.

The analyses were conducted in triplicate, and the validation parameters are presented in Table S4.
The table reports linearity range, Limit of detection and limit of quantification for each analyte, based on the calibration curve construction.

Table S5. Linearity, LOD and LOQ for PFASs.

	linearity range ug/L	LOD	LOQ
13C4_PFBA	0.1-250	0.01	0.03
13C5_PFPeA	0.1-250	0.01	0.03
13C2_PFHxA	0.1-250	0.01	0.03
13C4_PFHpA	0.1-250	0.01	0.03
13C4_PFOA	0.1-250	0.01	0.03
13C5_PFNA	0.1-250	0.01	0.03
13C2_PFDA	0.1-250	0.01	0.03
13C2_PFUdA	0.1-250	0.01	0.03
13C2_PFDoA	0.1-250	0.01	0.03
13C4_PFOS	0.1-250	0.01	0.03
d3MeFOSA	0.1-250	0.01	0.03

PAHs chromatographic conditions

Inlet conditions:

Heater	180°C
Pressure	18.634 psi
Septum purge flow	3 ml/min
Septum purge flow mode	standard
Mode	splitless
Purge flow to split vent	40 ml/min at 2 min

	Rate (°C/min)	Temperature (°C)	Hold time (min)	Run time (min)
Initial		180	0,005	0.005
Ramp 1	600	300	0	62,905

Oven conditions:

	Rate (°C/min)	Temperature (°C)	Hold time (min)	Run time (min)
Initial		120	1	1
Ramp 1	15	210	0	7
Ramp 2	22	340	50	62.909

Oven temperature	120°C
Equilibration time	0 min
Maximum oven temperature	350°C
Post run	130°C

PAHs analyzed are listed in Tab.1

rt	analite	precursor	product	Dwell	collision energy
7	naftalene	128	127	25	20
	naftalene	128	102	25	22
9,5	fluorene	166	165	15	30
	acenaftene	154	152	25	40
	acenaftene	153	152	25	40
	acenaftilene	152	151	25	40
	acenaftilene	152	150	25	40
12,5	antracene	178	176	15	34
	phenantrene	178	176	15	34
14	fluorantene	202	201	15	30
	pyrene	202	201	15	30
	fluorantene	202	200	15	50
	pyrene	202	200	15	45
15,5	perylene	252	250	75	42
	benzo_a_pirene	252	250	75	42
	benzo_b,j_fluorantene	252	250	75	42
	benzo_e_pirene	252	250	75	42
	benzo_k_fluorantene	252	250	75	42
	benzo_b,j_fluorantene	250	248	75	40
	perylene	250	248	75	40
	benzo_a_pirene	250	248	75	40
	benzo_k_fluorantene	250	248	75	40
	benzo_e_pirene	250	248	75	40
	benzo_a_antracene	228	226	40	38
	crisene	228	226	40	38
21	dibenzo_ah_antracene	278	276	75	38
	indeno 1,2,3 cd pirene	276	274	75	42
	benzo_ghi_perylene	276	274	75	38
26	dibenzo_a,e_pyrene	302	300	75	50
	dibenzo_a,l_pyrene	302	300	75	50
	dibenzo_ah_pyrene	302	300	75	50

	dibenzo_a,i_pyrene	302	300	75	50

Tab 1 PAHs analyzed

Dioxins chromatographic conditions

Inlet conditions:

Heater	130°C
Pressure	19.344 psi
Septum purge flow	3 ml/min
Septum purge flow mode	standard
Mode	splitless
Purge flow to split vent	40 ml/min at 2 min

	Rate (°C/min)	Temperature (°C)	Hold time (min)	Run time (min)
Initial		130	0,005	0.005
Ramp 1	600	300	0	65

Oven conditions:

	Rate (°C/min)	Temperature (°C)	Hold time (min)	Run time (min)
Initial		130	2	2
Ramp 1	10	200	16	25
Ramp 2	5	235	7	39
Ramp 3	5	340	5	65

Oven temperature	130°C
Equilibration time	0 min
Maximum oven temperature	350°C
Post run	130°C

PCBs chromatographic conditions

Inlet conditions:

Heater	106°C
Pressure	21.053 psi
Septum purge flow	3 ml/min
Septum purge flow mode	Standard
Mode	Splitless
Purge flow to split vent	40 ml/min at 2 min

	Rate (°C/min)	Temperature (°C)	Hold time (min)	Run time (min)
Initial		106	0,005	0.005
Ramp 1	600	325	0	57

Oven conditions:

	Rate (°C/min)	Temperature (°C)	Hold time (min)	Run time (min)
Initial		80	3	3
Ramp 1	20	160	0	7
Ramp 2	4	300	15	57

Oven temperature	80°C
Equilibration time	0 min
Maximum oven temperature	350°C
Post run	130°C

The PCBs detected are listed in Tab.3

**rt**	**analite**	**precursor**	**product**	**Dwell**	**collision energy**
16	epta_pcb183	393,9	323,9	20	35
	epta_pcb177	393,9	323,9	20	35

	epta_pcb187	393,9	323,9	20	35
	epta_pcb189	393,9	323,9	20	35
	epta_pcb180	393,9	323,9	20	35
	epta_pcb170	393,9	323,9	20	35
	epta_pcb183	391,9	321,9	20	35
	epta_pcb187	391,9	321,9	20	35
	epta_pcb177	391,9	321,9	20	35
	epta_pcb170	391,9	321,9	20	35
	epta_pcb189	391,9	321,9	20	35
	epta_pcb180	391,9	321,9	20	35
	esa_pcb167	359,9	289,9	20	28
	esa_pcb157	359,9	289,9	20	28
	esa_pcb138	359,9	289,9	20	28
	esa_pcb169	359,9	289,9	20	28
	esa_pcb156	359,9	289,9	20	28
	esa_pcb149	359,9	289,9	20	28
	esa_pcb146	359,9	289,9	20	28
	esa_pcb128	359,9	289,9	20	28
	esa_pcb153	359,9	289,9	20	28
	esa_pcb151	359,9	289,9	20	28
	esa_pcb138	357,9	287,9	20	28
	esa_pcb167	357,9	287,9	20	28
	esa_pcb153	357,9	287,9	20	28
	esa_pcb151	357,9	287,9	20	28
	esa_pcb128	357,9	287,9	20	28
	esa_pcb156	357,9	287,9	20	28
	esa_pcb149	357,9	287,9	20	28
	esa_pcb146	357,9	287,9	20	28
	esa_pcb157	357,9	287,9	20	28
	esa_pcb169	357,9	287,9	20	28
	13C_TCDD	333,9	269,9	20	24
	13C_TCDD	331,9	267,9	20	24

	penta_pcb101	325,9	255,9	20	28
	penta_pcb95	325,9	255,9	20	28
	penta_pcb126	325,9	255,9	20	28
	penta_pcb123	325,9	255,9	20	28
	penta_pcb105	325,9	255,9	20	28
	penta_pcb118	325,9	255,9	20	28
	penta_pcb110	325,9	255,9	20	28
	penta_pcb99	325,9	255,9	20	28
	penta_pcb114	325,9	255,9	20	28
	penta_pcb110	323,9	253,9	20	28
	penta_pcb105	323,9	253,9	20	28
	penta_pcb99	323,9	253,9	20	28
	penta_pcb126	323,9	253,9	20	28
	penta_pcb114	323,9	253,9	20	28
	penta_pcb101	323,9	253,9	20	28
	penta_pcb123	323,9	253,9	20	28
	penta_pcb118	323,9	253,9	20	28
	penta_pcb95	323,9	253,9	20	28
	tetra_pcb81	291,9	221,9	20	28
	tetra_pcb77	291,9	221,9	20	28
	tetra_pcb52	291,9	221,9	20	24
	tetra_pcb44	291,9	221,9	20	24
	tetra_pcb81	289,9	219,9	20	28
	tetra_pcb52	289,9	219,9	20	24
	tetra_pcb77	289,9	219,9	20	24
	tetra_pcb44	289,9	219,9	20	24
	tri_pcb31	258	186	20	24
	tri_pcb28	258	186	20	24
	tri_pcb18	258	186	20	24
	tri_pcb31	256	186	20	24
	tri_pcb18	256	186	20	24
	tri_pcb28	256	186	20	24

Table S6. Summary statistics (mean ± SD, percentiles, min–max) for target PAH in wild boar tissues. Values in µg/kg

	Matrix	Mean ± SD	Min - Max	Percentile 25th	Percentile 50th	Percentile 75th	p value	DF (%)
Naftalene	Muscle	0.084 ± 0.049	0 - 0.13	0.075	0.11	0.12	< .001	76.923
	Liver	1.139 ± 0.625	0 - 2.03	0.99	1.35	1.47		81.081
Acenaftilene	Muscle			N.D.			< .001	0
	Liver	0.232 ± 0.125	0 - 0.5	0.21	0.26	0.29		83.784
Acenaftene	Muscle	0.055 ± 0.015	0.01 - 0.08	0.05	0.05	0.07	< .001	100
	Liver			N.D.				0
Fluorene	Muscle			N.D.			< .001	0
	Liver	0.327 ± 0.145	0 - 0.73	0.31	0.35	0.4		89.189
Antracene	Muscle	0.248 ± 0.079	0 - 0.33	0.24	0.27	0.29	< .001	92.308
	Liver	1.813 ± 0.914	0 - 5.32	1.59	1.8	2.11		89.189
Phenantrene	Muscle			N.D.				0
	Liver							
Pyrene	Muscle			N.D.			< .001	0
	Liver	3.826 ± 2.934	0 - 7.6	0	5.57	6.19		64.865
Fluorantene	Muscle	0.293 ± 0.145	0 - 0.59	0.215	0.25	0.435	< .001	94.872
	Liver	3.653 ± 1.369	0 - 7.44	3.36	3.8	4.25		94.595
Benzo_a_antracene	Muscle			N.D.				
	Liver							
Crisene	Muscle			N.D.				
	Liver							
Benzo_a_pyrene	Muscle			N.D.				
	Liver							
Perylene	Muscle			N.D.				
	Liver							
Benzo_e_pirene	Muscle			N.D.			< .001	0
	Liver	0.674 ± 0.393	0 - 1.16	0.67	0.85	0.9		78.378
Benzo_k_fluorantene	Muscle			N.D.			< .001	0
	Liver	0.047 ± 0.025	0 - 0.09	0.04	0.05	0.06		81.081
Benzo_b,j_fluorantene	Muscle			N.D.			< .001	0
	Liver	0.023 ± 0.014	0 - 0.04	0.01	0.03	0.03		78.378
Dibenzo_a,e_pyrene	Muscle			N.D.				
	Liver							
Dibenzo_ah_pyrene	Muscle			N.D.				
	Liver							
Benzo_ghi_perylene	Muscle			N.D.				
	Liver							
Indeno 1,2,3 cd pyrene	Muscle			N.D.				
	Liver							
Dibenzo_a,i_pyrene	Muscle			N.D.				
	Liver							

Dibenzo_a,l_pyrene	Muscle<br>Liver	N.D.
Dibenzo_ah_antracene	Muscle<br>Liver	N.D.

Table S7. Summary statistics (mean ± SD, percentiles, min–max) for target PAH in wild boar tissues. Values in µg/kg

	Matrix	Media	Min - Max	Percentile 25th	Percentile Median	Percentile 75th	pvalue	DF (%)
**Naftalene**	Muscle	0.758 ± 0.457	0 - 2.15	0.665	0.76	0.865	< .001	89.474
	Liver	3.49 ± 3.085	0 - 18.43	2.59	3.28	4.18		83.784
**Acenaftilene**	Muscle	0.076 ± 0.346	0 - 2.14	0.01	0.01	0.01	< .001	84.211
	Liver	0.166 ± 0.101	0 - 0.49	0.15	0.16	0.193		86.486
**Acenaftene**	Muscle	0.006 ± 0.039	0 - 0.24	0	0	0	0.337	2.632
	Liver	N.D.						0.000
**Fluorene**	Muscle	0.124 ± 0.290	0 - 1.41	0.05	0.06	0.07	< .001	86.842
	Liver	0.279 ± 0.167	0 - 0.62	0.24	0.28	0.38		81.081
**Antracene**	Muscle	0.867 ± 0.531	0 - 2.25	0.36	1.105	1.178	< .001	84.211
	Liver	1.641 ± 0.972	0 - 3.39	0.65	2.01	2.19		83.784
**Phenantrene**	Muscle	0.01 ± 0.046	0 - 0.25	0	0	0	0.166	5.263
	Liver	N.D.						0.000
**Pyrene**	Muscle	1.075 ± 0.831	0 - 4.25	0.983	1.08	1.228	< .001	81.579
	Liver	9.294 ± 7.048	0 - 35.31	5.92	9.35	11.29		81.081
**Fluorantene**	Muscle	0.159 ± 0.321	0 - 1.36	0.063	0.08	0.08	< .001	76.316
	Liver	7.361 ± 5.406	0 - 22.32	4.8	7.39	8.81		81.081
**Benzo_a_antracene**	Muscle<br>Liver	N.D.						
**Crisene**	Muscle<br>Liver	N.D.						
**Benzo_a_pirene**	Muscle<br>Liver	N.D.						
**Perylene**	Muscle<br>Liver	N.D.						
**Benzo_e_pirene**	Muscle	0.009 ± 0.032	0 - 0.14	0	0	0	< .001	7.895
	Liver	0.866 ± 0.595	0 - 2.71	0.71	0.92	1.15		81.081
**Benzo_k_fluorantene**	Muscle<br>Liver	N.D.						
**Benzo_b.j_fluorantene**	Muscle<br>Liver	N.D.						
**Dibenzo_a.e_pyrene**	Muscle<br>Liver	N.D.						
**Dibenzo_ah_pyrene**	Muscle<br>Liver	N.D.						
**Benzo_ghi_perylene**	Muscle	N.D.						

	Liver	
**Indeno 1,2,3 cd pirene**	Muscle<br>Liver	N.D.
**Dibenzo_a.i_pyrene**	Muscle<br>Liver	N.D.
**Dibenzo_a.l_pyrene**	Muscle<br>Liver	N.D.
**Dibenzo_ah_antracene**	Muscle<br>Liver	N.D.

Table S8. Mean ± SD, median with percentile and ranges of target PCB in liver and muscle of wild boar. Values are reported in μg/kg.

				Percentile				
	**Matrix**	**Mean ± SD**	**Min - Max**	**25th**	**Median**	**75th**	**pvalue**	**DF%**
**PCB 31**	Liver	4.045 ± 3.008	0 - 7.867	0	5.533	6.203	< .001	66.667
	Muscle	11.689 ± 7.088	0 - 30.818	7.951	12.622	14.238		170.940
**PCB 28**	Liver	N.D.					< .001	
	Muscle	15.943 ± 11.619	0 - 45.614	8.406	17.722	19.615		76.923
**PCB 18**	Liver	0.037 ± 0.018	0 - 0.056	0.034	0.046	0.050	< .001	82.051
	Muscle	11.504 ± 6.673	0 - 27.826	9.926	12.63	14.478		82.051
**PCB 81**	Liver	N.D.					< .001	0
	Muscle	2.078 ± 1.222	0 - 6.156	1.606	2.062	2.417		87.179
**PCB 77**	Liver	0.235 ± 0.094	0 - 0.314	0.243	0.265	0.285	< .001	87.179
	Muscle	0.836 ± 0.483	0 - 2.291	0.637	0.809	0.963		92.308
**PCB 99**	Liver	N.D.					< .001	0
	Muscle	0.668 ± 0.382	0 - 1.576	0.505	0.643	0.787		89.744
**PCB 95**	Liver	N.D.					< .001	0
	Muscle	0.188 ± 0.081	0 - 0.499	0.165	0.179	0.189		97.436
**PCB 126**	Liver	0.205 ± 0.094	0 - 0.299	0.193	0.231	0.266	< .001	84.615
	Muscle	0.395 ± 0.247	0 - 1.169	0.309	0.383	0.466		89.744
**PCB 52**	Liver	N.D.					< .001	0
	Muscle	0.148 ± 0.068	0 - 0.366	0.131	0.144	0.168		92.3077
**PCB 123**	Liver	N.D.					< .001	0
	Muscle	0.154 ± 0.089	0 - 0.419	0.113	0.146	0.178		92.308
**PCB 44**	Liver	N.D.					< .001	0
	Muscle	0.376 ± 0.199	0 - 0.980	0.300	0.3576	0.406		97.436
**PCB 167**	Liver	N.D.					< .001	0
	Muscle	0.137 ± 0.075	0 - 0.361	0.113	0.137	0.151		97.436
**PCB 157**	Liver	0	0	0	0	0	< .001	0
	Muscle	0.442 ± 0.217	0 - 1.075	0.390	0.452	0.513		94.872
**PCB 118**	Liver<br>Muscle	N.D.						
**PCB 114**	Liver<br>Muscle	N.D.						
**PCB 110**	Liver	N.D.						

	Muscle							
**PCB 156**	Liver	N.D.					< .001	0
	Muscle	0.651 ± 0.222	0.224 - 1.426	0.575	0.635	0.685		100
**PCB 153**	Liver	N.D.					< .001	0
	Muscle	0.167 ± 0.099	0 - 0.502	0.134	0.163	0.190		89.744
**PCB 105**	Liver	N.D.						
	Muscle							
**PCB 151**	Liver	N.D.						
	Muscle							
**PCB 101**	Liver	N.D.						
	Muscle							
**PCB 189**	Liver	N.D.						
	Muscle							
**PCB 187**	Liver	N.D.					< .001	0
	Muscle	1.669 ± 1.117	0 - 4.024	1.034	1.756	2.110		82.051
**PCB 149**	Liver	N.D.						
	Muscle							
**PCB 146**	Liver	N.D.						
	Muscle							
**PCB 183**	Liver	N.D.						
	Muscle							
**PCB 138**	Liver	N.D.						
	Muscle							
**PCB 128**	Liver	N.D.						
	Muscle							
**PCB 180**	Liver	0.224 ± 0.252	0 - 0.669	0	0	0.438	0.316	46.154
	Muscle	0.312 ± 0.201	0 - 0.907	0.232	0.3	0.368		87.179
**PCB 169**	Liver	N.D.						
	Muscle							
**PCB 177**	Liver	N.D.						
	Muscle							
**PCB 170**	Liver	N.D.					< .001	0
	Muscle	1.973 ± 1.390	0 - 6.242	1.498	2.209	2.416		76.923

Table S9. Mean ± SD, median with percentile and ranges of target PCB in liver and muscle of swine. Values are reported in µg/kg.

				Percentile				
	**Matrix**	**Mean ± SD**	**Min - Max**	**25th**	**Median**	**75th**	**pvalue**	**DF%**
**PCB 31**	Muscle	2.696 ± 1.265	0 - 5.67	1.915	2.84	3.675	< .001	92.308
	Liver	0.52 ± 0.315	0 - 1.02	0.28	0.495	0.828		94.737
**PCB 28**	Muscle	5.896 ± 4.169	0 - 18.15	4.69	6.1	7.545	< .001	76.923
	Liver	N.D.						0
**PCB 18**	Muscle	3.971 ± 1.114	0 - 7.67	3.57	3.97	4.275	< .001	97.436
	Liver	0.048 ± 0.009	0 - 0.06	0.05	0.05	0.05		97.368

**PCB 81**	Muscle	1.529 ± 1.011	0 - 4.5	1.125	1.63	1.935	< .001	82.051
	Liver	N.D.						0
**PCB 77**	Muscle	0.7903 ± 0.349	0 - 1.43	0.71	0.8	0.98	< .001	89.744
	Liver	0.233 ± 0.106	0 - 0.32	0.225	0.28	0.29		84.211
**PCB 99**	Muscle	0.594 ± 0.351	0 - 1.64	0.45	0.63	0.8	< .001	84.615
	Liver	N.D.						0
**PCB 95**	Muscle	0.089 ± 0.052	0 - 0.28	0.07	0.09	0.11	< .001	89.744
	Liver	N.D.						0
**PCB 126**	Muscle	0.238 ± 0.172	0 - 0.9	0.14	0.23	0.31	0.943	84.615
	Liver	0.219 ± 0.068	0 - 0.28	0.2225	0.24	0.25		92.105
**PCB 52**	Muscle	0.104 ± 0.043	0 - 0.26	0.09	0.11	0.12	< .001	92.308
	Liver	N.D.						0
**PCB 123**	Muscle	0.102 ± 0.051	0.01 - 0.27	0.085	0.1	0.12	< .001	89.744
	Liver	N.D.						0
**PCB 44**	Muscle	0.331 ± 0.156	0 - 0.84	0.275	0.32	0.4	< .001	92.308
	Liver	N.D.						0
**PCB 167**	Muscle	0.089 ± 0.038	0 - 0.16	0.08	0.09	0.1	< .001	94.872
	Liver	N.D.						0
**PCB 157**	Muscle	0.033 ± 0.014	0 - 0.07	0.03	0.03	0.04	< .001	92.308
	Liver	N.D.						0
**PCB 118**	Muscle	N.D.						
	Liver							
**PCB 114**	Muscle	N.D.						
	Liver							
**PCB 110**	Muscle	N.D.						
	Liver							
**PCB 156**	Muscle	0.531 ± 0.307	0 - 1.49	0.41	0.52	0.61	< .001	97.436
	Liver	N.D.						0
**PCB 153**	Muscle	N.D.						
	Liver							
**PCB 105**	Muscle	N.D.						
	Liver							
**PCB 151**	Muscle	N.D.						
	Liver							
**PCB 101**	Muscle	N.D.						
	Liver							
**PCB 189**	Muscle	N.D.						
	Liver							
**PCB 187**	Muscle	1.128 ± 0.430	0 - 2.23	0.95	1.17	1.32	< .001	97.436
	Liver	0.329 ± 0.459	0 - 1.33	0	0	0.87		36.842
**PCB 149**	Muscle	N.D.						
	Liver							
**PCB 146**	Muscle	N.D.						
	Liver							
**PCB 183**	Muscle	N.D.						
	Liver							
**PCB 138**	Muscle	N.D.						

	Liver							
**PCB 128**	Muscle<br>Liver	N.D.						
**PCB 180**	Muscle	0.199 ± 0.077	0.02 - 0.42	0.16	0.19	0.215	0.232	100
	Liver	0.130 ± 0.108	0 - 0.26	0	0.2	0.21		60.526
**PCB 169**	Muscle<br>Liver	N.D.						
**PCB 177**	Muscle<br>Liver	N.D.						
**PCB 170**	Muscle	1.697 ± 1.151	0 - 4.42	1.21	1.83	2.175	< .001	79.487
	Liver	0.2295 ± 0.598	0 - 1.9	0	0	0		13.158

Table S10. Mean ± SD, median with percentile and ranges of target PFAS in liver and muscle of wild boar. Values are reported in µg/kg.

				**Percentile**				
	**Matrix**	**Mean ± SD**	**Min - Max**	**25th**	**Median**	**75th**	**pvalue**	**DF (%)**
**PFBA**	liver	136.247 ± 189.297	0.01 - 680.282	7.000	61.226	185.1313	0.032	100
	muscle	23.608 ± 49.374	0.01 - 179.662	1.976	5.351	12.297		100
**PFPeA**	liver<br>muscle	< LOQ						100<br>100
**PFHxA**	liver	3.191 ± 3.308	0.088 - 9.377	0.503	1.372	6.240	0.493	100
	muscle	2.607 ± 3.812	0.024 - 12.301	0.234	0.826	4.186		100
**PFHpA**	liver	56.783 ± 99.729	6.9113 - 397.112	17.970	30.509	48.939	< .001	100
	muscle	10.130 ± 4.775	3.054 - 20.362	6.730	9.999	12.712		100
**PFOA**	liver	424.370 ± 438.077	31.335 - 1504.985	99.445	292.4903	545.4161	< .001	100
	muscle	42.650 ± 44.605	10.323 - 180.455	18.757	26.799	37.835		100
**PFNA**	liver	208.616 ± 166.745	26.989 - 593.141	71.521	189.262	301.8965	< .001	100
	muscle	6.909 ± 4.753	1.414 - 19.4518	4.690	6.098	7.289		100
**PFDA**	liver	157.963 ± 177.841	26.072 - 603.357	35.768	86.622	201.336	< .001	100
	muscle	4.277 ± 3.57	0.518 - 15.288	2.359	3.518	5.094		100
**PFUdA**	liver	96.083 ± 136.718	9.103 - 437.891	17.819	30.186	108.88	< .001	100
	muscle	2.9552 ± 2.3916	0.378 - 8.330	1.057	1.797	4.350		100
**PFDoA**	liver	84.858 ± 149.844	2.774 - 498.632	9.163	12.938	85.087	< .001	100
	muscle	1.961 ± 1.731	0.284 - 5.921	0.844	1.166	2.743		100
**PFTrDA**	liver	154.306 ± 292.098	1.715 - 926.597	4.991	20.271	132.8584	< .001	100
	muscle	2.056 ± 2.626	0.290 - 10.611	0.573	0.985	2.134		100
**PFTeDA**	liver	34.794 ± 69.701	0.187 - 225.411	0.642	3.181	28.455	< .001	100
	muscle	0.484 ± 0.686	0.055 - 2.784	0.104	0.228	0.509		100
**PFHxDA**	liver	4.433 ± 9.954	0.010 - 34.644	0.033	0.049	2.478	0.002	100
	muscle	0.041 ± 0.074	0.003 - 0.318	0.01	0.02	0.026		100
**PFODA**	liver<br>muscle	< LOQ						100<br>100
**PFBS**	liver	0.440 ± 0.551	0.017 - 1.505	0.039	0.142	0.883	0.625	100
	muscle	0.284 ± 0.464	0.01 - 1.772	0.061	0.090	0.176		100

**PFHxS**	liver	208.090 ± 110.686	52.491 - 485.835	123.773	207.958	259.3407	< .001	100
	muscle	42.062 ± 29.379	8.476 - 120.058	17.640	36.433	56.497		100
**PFHpS**	liver	15.221 ± 16.663	2.319 - 56.727	5.288	9.091	12.187	0.769	100
	muscle	12.309 ± 11.609	1.796 - 44.284	3.937	9.597	15.365		100
**PFOS**	liver	< LOQ						100
	muscle							100
**PFDS**	liver	< LOQ						100
	muscle							100
**PFOSA**	liver	< LOQ						100
	muscle							100
**MeFOSA**	liver	< LOQ						100
	muscle							100
**EtFOSA**	liver	2.109 ± 2.278	0.012 - 5.678	0.154	0.809	4.527	0.077	100
	muscle	0.406 ± 0.577	0.014 - 2.214	0.110	0.144	0.307		100
**N-MeFOSAA**	liver	< LOQ						100
	muscle							100
**N-EtFOSAA**	liver	< LOQ						100
	muscle							100

Table S11. Mean ± SD, median with percentile and ranges of target PFAS in liver and muscle of swine. Values are reported in μg/kg.

				Percentile				
	**Matrix**	**Media**	**Minimo**	**25th**	**Median**	**75th**	**pvalue**	**DF (%)**
**PFBA**	liver	115.214 ± 86.849	4.256 - 322.080	72.370	103.246	138.467	< .001	100
	muscle	11.314 ± 10.448	0.01 - 27.924	1.778	7.482	23.065		100
**PFPeA**	liver	< LOQ						100
	muscle							100
**PFHxA**	liver	6.518 ± 16.320	0.138 - 63.430	0.543	1.302	2.492	0.244	100
	muscle	2.963 ± 4.234	0.084 - 11.516	0.291	0.563	5.847		100
**PFHpA**	liver	5.891 ± 7.989	1.438 - 33.477	2.592	3.488	4.926	0.108	100
	muscle	3.921 ± 3.948	0.462 - 11.698	1.064	1.982	6.979		100
**PFOA**	liver	78.211 ± 94.251	2.086 - 335.428	16.317	37.301	104.700	0.108	100
	muscle	24.427 ± 19.836	3.314 - 81.040	9.314	20.910	35.396		100
**PFNA**	liver	18.655 ± 41.295	2.618 - 166.665	5.846	6.910	9.033	< .001	100
	muscle	3.406 ± 1.815	0.838 - 6.262	1.976	3.474	5.187		100
**PFDA**	liver	3.331 ± 2.41	0.832 - 8.414	1.468	2.305	4.266	0.004	100
	muscle	1.489 ± 1.416	0.349 - 4.831	0.481	0.792	2.577		100
**PFUdA**	liver	2.629 ± 5.331	0.206 - 20.933	0.347	0.534	1.509	0.03	100
	muscle	0.642 ± 0.698	0.093 - 2.163	0.171	0.265	1.008		100
**PFDoA**	liver	1.291 ± 2.085	0.017 - 6.927	0.126	0.240	0.908	0.63	100
	muscle	0.426 ± 0.453	0.059 - 1.551	0.122	0.235	0.512		100
**PFTrDA**	liver	0.993 ± 1.849	0.044 - 6.166	0.110	0.135	0.290	0.361	100
	muscle	0.189 ± 0.174	0.006 - 0.671	0.083	0.108	0.288		100
**PFTeDA**	liver	0.483 ± 1.170	0.008 - 4.406	0.037	0.068	0.092	0.682	100
	muscle	0.087 ± 0.093	0.021 - 0.341	0.041	0.057	0.085		100

**PFHxDA**	liver	0.479 ± 1.719	0.001 - 6.687	0.005	0.008	0.025	0.126	100
	muscle	0.025 ± 0.019	0.006 - 0.067	0.011	0.015	0.032		100
**PFODA**	liver	1.291 ± 2.085	0.017 - 6.927	0.126	0.240	0.908	0.63	100
	muscle	0.426 ± 0.453	0.059 - 1.551	0.122	0.235	0.512		100
**PFBS**	liver	1.093 ± 1.219	0.004 - 4.147	0.031	0.661	1.912	0.274	100
	muscle	0.392 ± 0.587	0.004 - 2.334	0.081	0.172	0.327		100
**PFHxS**	liver	311.489 ± 315.703	95.208 - 1401.28	156.254	217.754	303.530	< .001	100
	muscle	76.412 ± 58.136	12.447 - 201.525	34.012	55.132	99.195		100
**PFHpS**	liver	27.155 ± 54.162	4.777 - 202.771	5.825	8.176	11.197	0.656	100
	muscle	9.40 ± 6.743	1.334 - 24.235	3.937	8.633	12.841		100
**PFOS**	liver	< LOQ						100
	muscle							100
**PFDS**	liver	< LOQ						100
	muscle							100
**PFOSA**	liver	< LOQ						100
	muscle							100
**MeFOSAA**	liver	< LOQ						100
	muscle							100
**EtFOSAA**	liver	1.254 ± 3.495	0.023 - 13.743	0.069	0.146	0.459	0.259	100
	muscle	0.139 ± 0.131	0.01 - 0.455	0.050	0.086	0.156		100
**N-MeFOSAA**	liver	< LOQ						100
	muscle							100
**N-EtFOSAA**	liver	< LOQ						100
	muscle							100
